# Carbon nanotube promotes contraction and electrical activity of neonatal cardiomyocytes by targeting sodium/calcium exchanger NCX1

**DOI:** 10.1038/s41392-023-01397-5

**Published:** 2023-05-05

**Authors:** Xiao Zhang, Yuan Shen, Zhen Cao, Ying Zhu, Wei Liu, Wei Wu, Chunlan Wang, Siwei Li, Jin Zhou, Changyong Wang

**Affiliations:** Beijing Institute of Basic Medical Sciences, 27 Taiping Rd, Beijing, 100850 China

**Keywords:** Cell biology, Nanobiotechnology

**Dear Editor**,

The conductive nanomaterials, such as carbon nanotubes (CNTs), have attracted much attention in biomedical applications. Particularly, it has been found that CNTs can promote the electrical activity of cardiomyocytes, but little is known about the mechanism.^1,2^ To better understand the CNTs’ biological effects on cardiomyocytes and the underlying mechanism, we carried out researches from three aspects: (1) we tested phenotype changes in cardiomyocytes treated with CNTs; (2) we studied changes of protein expression profiles in cardiomyocytes treated with CNTs using high-throughput proteomics technology; (3) Based on the results of proteomics analysis, we explored the molecular mechanism of CNTs regulating the phenotype changes of cardiomyocytes via gene manipulation.

Before exploring the effects of CNTs on cardiomyocytes, we analyzed the basic physicochemical properties and cytotoxicity of CNTs. Commercial multi-walled carbon nanotubes (MWCNTs) were measured with diameter of 20–30 nm, length of 10–30 μm (Supplementary Fig. 1a) and characterized D and G bands of the graphitic carbon structures (Supplementary Fig. 1b). Cell counting Kit 8 (CCK-8) analysis showed MWCNTs are not toxic to NRVMs at or below the concentration of 30 μg/mL in the cell culture medium (Supplementary Fig. 1c). These results indicated that MWCNTs can be used to study the effects of nanomaterials on cardiomyocytes at the proper concentration.

On this basis, to test phenotype changes in cardiomyocytes treated with CNTs, we studied the effects of MWCNTs on neonatal rat ventricular cardiomyocytes (NRVMs) in typical structure and physiological characteristics and function.

To investigate the effects of MWCNTs on the typical structure of NRVMs, we studied the distribution of MWCNTs in cardiomyocytes and then systematically analyzed expression of structure-specific genes of NRVMs. TEM and hematoxylin and eosin (H&E) staining results showed an intracellular distribution of MWCNTs, which could still be observed at 10 d (Supplementary Fig. 2a, b). These results suggested that MWCNTs could be absorbed in NRVMs and remain intracellular for a period of time. Immunofluorescence results of structural protein (cTnT) and intercalated discs-related proteins (Cx43, Plakoglobin, Desmoplakin and N-cadherin) showed that MWCNTs treatment induced the formation of better organized junctions at cell borders (Fig. 1a). Quantitative reverse transcription polymerase chain reaction (qRT-PCR) and western blot analysis also confirmed that MWCNTs treatment caused upregulation of these proteins in intercalated discs (Fig. 1b, c). These results implied that MWCNTs were absorbed and involved in the formation of better cell junctions of NRVMs, which may enable cardiomyocytes to possess stronger electrical activity and cardiac contraction function.

**Fig. 1:**
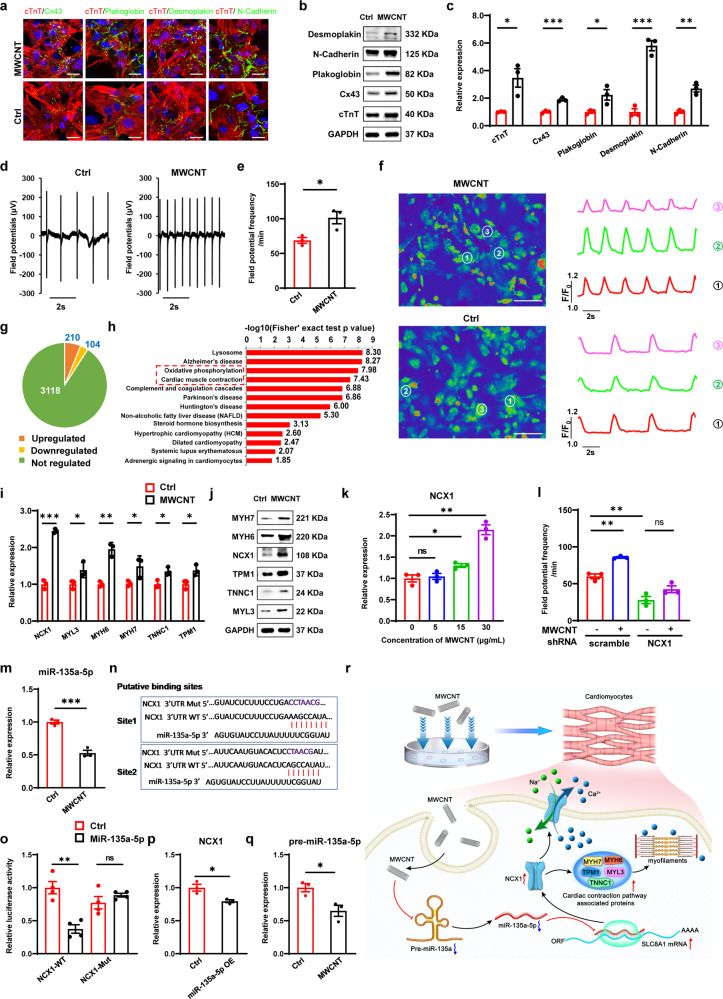
Carbon nanotube promotes contraction and electrical activity of neonatal cardiomyocytes by targeting sodium/calcium exchanger NCX1. **a** Immunofluorescence images of structural protein (cTnT) and intercalated discs-related proteins (Cx43, Plakoglobin, Desmoplakin and N-cadherin) of NRVMs with MWCNTs treatment for 10 days. Connexin-43 (green); plakoglobin (green); Desmoplakin (green); N-Cadherin (green); cTnT (red); DNA (DAPI); Scale bars, 20 μm. **b**, **c** Expression level of structural protein (cTnT) and intercalated discs-related proteins (Cx43, Plakoglobin, Desmoplakin and N-cadherin) of NRVMs by western blot (**b**) and qRT-PCR analysis (**c**). *n* = 3. **d**, **e** MEA assay of NRVMs with MWCNTs treatment for 10 days. Electrical signal recording curves (**d**) and statistical evaluation of field potential frequency (**e**). *n* = 3. **f** Calcium transient (left) and calcium signal recording curves (right) of NRVMs with MWCNTs treatment for 10 days. F/F_0_ refers to the measured fluorescence normalized to background fluorescence. Scale bars: 50 μm. **g** Comprehensive quantitative proteomics data. 3432 proteins were identified and 210 proteins upregulated and 104 proteins downregulated in MWCNTs treated NRVMs. **h** Top canonical pathways from KEGG pathway analysis with upregulated proteins. **i**, **j** Expression level of proteins enriched in cardiac muscle contraction pathway by qRT-PCR (**i**) and western blot analysis (**j**). *n* = 3. **k** NCX1 expression level at different concentrations of MWCNTs by qRT-PCR analysis. *n* = 3. **l** MEA assay of NRVMs with NCX1 knockdown and MWCNT treatment. *n* = 3. **m** qRT-PCR analysis of miR-135a-5p level. *n* = 3. **n** Putative binding sites of miR-135a-5p at NCX1 mRNA 3’UTR. **o** Dual luciferase reporter assay to verify binding interaction between NCX1 mRNA 3’UTR and miR-135a-5p in HEK293T cells. *n* = 4. **p** qRT-PCR analysis of NCX1 expression level in NRVMs after miR-135a-5p overexpression. *n* = 3. **q** qRT-PCR analysis of pre-miR-135a-5p level. *n* = 3. **r** Schematic representation of the proposed regulation of NCX1 by miR-135a-5p and pathways involved in MWCNTs-induced molecular events of NRVMs. Data are expressed as mean ± SEM. *P < 0.05, **P < 0.01, ***P < 0.001

To investigate the effects of MWCNTs on physiological characteristics and function of NRVMs, cardiac electrical and calcium activities were measured using multi-electrode microarray (MEA) and spontaneous calcium transient analysis. MEA recordings of extracellular field potentials showed MWCNTs increased spike frequency of NRVMs (Fig. 1d, e). Besides, we also tested the effects of MWCNTs on iPSC-derived cardiomyocytes (iPSC-CMs) by CCK-8 and MEA, and found similar results to NRVMs (Supplementary Fig. 3). Calcium transient analysis demonstrated NRVMs with MWCNTs treatment exhibited higher frequency and peak amplitude of spontaneous calcium transients (Fig. 1f and Supplementary Fig. 4a, b), implying NRVMs might possess stronger contractility. Further, to investigate the effects of MWCNTs on the contraction function of NRVMs, cell shortening assay was performed. The results showed that MWCNTs treatment enhanced cell shortening (Supplementary Fig. 4c), indicating stronger contraction capacity of cardiomyocytes. These results suggested that MWCNTs enhanced the electrical activity and contractile function of NRVMs.

Based on MWCNTs-induced enhancement in electrical activity and contraction function of NRVMs, to further investigate the molecular mechanism of MWCNTs regulating NRVMs, we studied changes of protein expression profiles in NRVMs treated with MWCNTs for 10 days using high-throughput proteomics technology (Supplementary Fig. 5a). We identified differentially expressed proteins in MWCNTs treated versus untreated cardiomyocytes (210 upregulated and 104 downregulated) (Fig. 1g and Tables S1, S2). Based on proteomics results, Kyoto Encyclopedia of Genes and Genomes (KEGG) and Gene Ontology (GO) analyses were performed. KEGG analysis showed that cardiac muscle contraction pathway was significantly upregulated (Fig. 1h), including three groups: (1) Myosin and tropomyosin (MYH7, MYL3, MYH6, TPM1), essential for sarcomere organization and myofibril assembly. (2) Troponins (TNNC1), associated with calcium ion binding and cardiac muscle contraction. (3) Sodium/calcium exchanger 1 (NCX1), a vital Ca^2+^ homeostasis regulator in cardiomyocytes. Oxidative phosphorylation pathway was also significantly upregulated, including ATP synthase, NADH dehydrogenase and cytochrome oxidase/reductase in the mitochondrial respiratory chain. Gene Ontology (GO) analysis also revealed that upregulated proteins were enriched in mitochondrial membrane component and calcium ion binding activity (Supplementary Fig. 5b). In addition, to verify the effects of MWCNTs on the typical structure and physiological characteristics and function of NRVMs, we analyzed changes in the expression of related proteins. The results showed that the expression levels of many proteins related to cell junctions, cardiac contraction and calcium handling were up-regulated after the treatment of MWCNTs (Supplementary Fig. 6a–c). These results suggested that MWCNTs may regulate the electrical activity and contraction function of NRVMs by affecting cardiac muscle contraction pathway, oxidative phosphorylation pathway and typical structural and calcium handling-related proteins.

Based on the previous physiological characteristics and function studies of NRVMs, we chose cardiac muscle contraction pathway for in-depth study. We used qRT-PCR and western blot to verify the expression changes of major genes in this pathway. The results showed that MWCNTs treatment significantly increased the expression levels of NCX1, MYL3, MLY7, MYH6, TNNC1 and TPM1 genes (Fig. 1i, j). In view of the important role of NCX1 in calcium handling and excitation-contraction coupling in cardiomyocytes,^3^ we conducted an in-depth study on NCX1. Firstly, to explore the regulation of MWCNT on NCX1 gene expression, we conducted a concentration gradient treatment experiment of MWCNTs. The results showed that gene expression level of NCX1 increased with the increase of MWCNTs concentration in a dose-dependent manner (Fig. 1k). Secondly, to better understand the mechanism of NCX1 in the regulation of contraction function and electrical activity of NRVMs by MWCNTs, we further explored the physiological function of NCX1. Based on that NCX1 was reported to regulate the expression of cardiac contraction-related genes by influencing calcium handling,^4^ we manipulated the expression level of NCX1 in NRVMs to detect the effects of NCX1 knockdown/overexpression on gene expression levels of MYL3, MYH6, MYH7, TNNC1 and TPM1. qRT-PCR analysis showed that NCX1 knockdown reduced the expression levels of these genes (Supplementary Fig. 7a, b), while overexpression of NCX1 showed the opposite results (Supplementary Fig. 7c, d). Further, to explore whether NCX1 mediated the regulation of MWCNTs on cardiac contraction, we examined the effects of MWCNTs on cardiac contraction function and related gene expression in NCX1 knockdown NRVMs. Cell shortening analysis showed that NCX1 knockdown abolished MWCNTs-induced enhancement of cell shortening (Supplementary Fig. 7e). qRT-PCR analysis showed NCX1 knockdown abolished MWCNTs-induced enhancement of many cardiac contraction-related gene expression (Supplementary Fig. 7b). These results suggested that NCX1 may mediate MWCNTs’ beneficial effects on cardiac contraction in NRVMs. To investigate whether NCX1 also mediated the effects of MWCNTs on electrical activity and calcium activity of cardiomyocytes, we examined the effects of MWCNTs on the electrical activity and calcium activity of cardiomyocytes in NCX1 knockdown NRVMs. The results showed that NCX1 knockdown abolished MWCNT-induced increase in firing frequency (Fig. 1l) and Ca^2+^ transient frequency (Supplementary Fig. 7f). These findings suggested that MWCNTs’ beneficial effects on cardiac contraction and electrical activity of NRVMs is dependent on NCX1.

In order to further explore the molecular mechanism of NCX1-mediated MWCNTs’ effects on NRVMs, we studied the pathways of NCX1. Inspired by that miR-135a was reported to regulate the electrical activity of cardiomyocytes by affecting the expression of NCX1,^5^ we screened the miRNAs targeting NCX1 and identified four miRNAs, including miR-135a-5p, miR-1b-3p, miR-9a-5p and miR-495-3p (Supplementary Fig. 8). To explore whether these miRNAs were involved in MWCNTs’ effects on NRVMs, we examined miRNAs expression levels under MWCNTs treatment. qRT-PCR analysis showed that MWCNTs treatment significantly reduced the expression levels of miR-135a-5p, miR-1b-3p and miR-9a-5p (Fig. 1m, Supplementary Fig. 9a, b). Combining previous reports and our experimental results, we selected miR-135a-5p for in-depth study. Firstly, we verified direct binding interaction between NCX1 mRNA 3’UTR and miR-135a-5p using dual luciferase reporter assay technique (Fig. 1n, o). Secondly, we manipulated the expression level of miR-135a-5p in NRVMs to study the effects of miR-135a-5p knockdown/overexpression on gene expression level of NCX1. qRT-PCR analysis showed that miR-135a-5p knockdown increased gene expression level of NCX1, while overexpression of miR-135a-5p showed the opposite results (Supplementary Fig. 10a–c and Fig. 1p). Thirdly, we examined the effects of miR-135a-5p knockdown on the electrical activity and contraction of NRVMs by MEA assay and cell shortening assay. The results showed that miR-135a-5p knockdown enhanced the electrical activity and contraction of NRVMs (Supplementary Fig. 11). Finally, to further explore whether miR-135a-5p was involved in MWCNTs’ effects on NRVMs, we tested the expression level of miR-135a-5p precursor (pre-miR-135a-5p). The results showed that MWCNTs treatment decreased the expression level of pre-miR-135a-5p (Fig. 1q). These results implied that miR-135a-5p might mediate the effects of MWCNTs on NRVMs by targeting NCX1.

In summary, our study demonstrated that carbon nanotube promotes contraction and electrical activity of NRVMs by increasing NCX1, and miRNA-135a-5p may be involved in these biological processes by targeting NCX1 (Fig. 1r). These findings provide new insights into the interaction between carbon nanotube and cardiomyocytes and lay a foundation for the application of conductive nanomaterials in the repair of myocardial injury.

## Supplementary information

Revised Supplementary Materials without highlight

## Data Availability

All data in this article and supplementary materials are publicly available to authorized users.
