# Supplementary material for: Carbon nanotube promotes contraction and electrical activity of neonatal cardiomyocytes by targeting sodium/calcium exchanger NCX1
Source: Signal Transduct Target Ther. 2023 May 5;8:180. doi: 10.1038/s41392-023-01397-5 (PMC10160085; doi:10.1038/s41392-023-01397-5)
Supplement: Supplementary file 1 — Revised Supplementary Materials without highlight [file 41392_2023_1397_MOESM1_ESM.docx]

Supplementary Materials for

Carbon nanotube promotes contraction and electrical activity of neonatal cardiomyocytes by targeting sodium/calcium exchanger NCX1

Xiao Zhangᶧ, Yuan Shenᶧ, Zhen Cao, Ying Zhu, Wei Liu, Wei Wu, Chunlan Wang, Siwei Li, Jin Zhou*, Changyong Wang*

Correspondence to: wcy2000_zm@163.com (Changyong Wang); sisun819@outlook.com (Jin Zhou)

**This PDF file includes:**

Materials and Methods

References

Figures. S1 to S11

Tables S1 to S3

Sequences of lentiviral NCX1 and miR-135a-5p

**SUPPLEMENTAL MATERIALS AND METHODS**

Preparation and characterization of MWCNTs

MWCNTs were commercially from Nanostructured & Amorphous Materials, Inc. (Houston, TX, USA) and samples were homogeneously dispersed in 0.1% Pluronic F-127 after sonication at 12 W for 5 min. TEM images were taken on Hitachi H 7500 electron microscope (Hitachi High Technologies, Tokyo, Japan) as reported previously.^1^ Raman spectra were performed with a Raman spectrometer (LabRAM HR Evolution, Horiba Scientific, Kyoto, Japan) as reported previously.^2^

NRVMs isolation and lentiviral transduction

NRVMs were isolated from 1-day-old Sprague-Dawley rats as reported previously.^3^ All of the animal experiments were performed following the guidelines of the Institutional Animal Care and Use Committee of the Chinese Academy of Military Medical Science and approved by its Committee on the Ethics of Animal Experiments. NRVMs (>90% purity) were immediately seeded at 2 h after pre-plating and were cultivated in DMEM (Invitrogen, Waltham, MA, USA) containing 15% FBS (Invitrogen, Waltham, MA, USA) at 37°C and 5% CO2. The culture medium was changed every 2 days. For NCX1/miR-135a-5p knockdown/overexpression, lentiviruses of NCX1 and miR-135a-5p were purchased from BrainVTA. NRVMs were treated with lentivirus of NCX1 or miR-135a-5p following standard protocol provided by BrainVTA. Briefly, NRVMs were cultured for 2 days and then treated with lentivirus of NCX1 or miR-135a-5p. NRVMs were replaced with fresh culture medium after 12 hours. After 2 days of lentivirus expression, NRVMs were treated with carbon nanotubes.

CCK-8 assay

Cell viability of NRVMs/iPSC-CMs was analyzed using the CCK-8 assay (Dojindo, CK04). NRVMs/iPSC-CMs were seeded into 24-well/96-well plates and MWCNTs were added 48 h later with a series of concentrations of 2，5，10，30，60，90 μg/mL. After 24 h, the medium was changed to 200 µL medium with CCK-8 (1:100) and incubated for 2 h. The detection wavelength was 450 nm in the microplate reader.

H&E staining

NRVMs were conducted with H&E staining according to the general procedure.^3^ The staining images were acquired with Nikon microscope (Tokyo, Japan, Ci-L) using 20× and 100× objectives.

Immunofluorescence staining

Cells were fixed in 4% paraformaldehyde for 30 min at room temperature and then permeabilized with 0.3% Triton X-100 in PBS for 10 min. After 1.5 h of prehybridization in PBS-2% bovine serum albumin and 0.05% sodium azide, the cells were incubated with the primary antibodies overnight at 4°C and the secondary fluorescence-conjugated antibodies for 30 min at room temperature. The following antibodies were used: mouse monoclonal anti-cTnT (Thermo Fisher Scientific, MA5-12960), anti-Connexin 43 (Abcam, ab11370), anti-N-cadherin (Abcam, ab18203), anti-desmoplakin I+II (Abcam, ab71690), and anti-Plakoglobin (Abcam, ab184919), Alexa Fluor 488-conjugated anti-rabbit IgG (Abcam, ab150077) and Alexa Fluor 568-conjugated anti-mouse IgG (Abcam, ab175702). Cells were counterstained with DAPI (Beyotime, C1005) and visualized under Zeiss confocal microscope (Oberkochen, Germany，LSM 880).

Transmission electronic microscope (TEM) analysis of NRVMs

TEM analysis of NRVMs was performed as reported previously.^4^ Cells were fixed sequentially with 2.5% of glutaraldehyde in 0.1 M sodium cacodylate buffer (pH 7.4) for 6 h and 1% of OsO4 in the phosphate-buffer for 2 h. After cells were embedded in the epoxy resin, semi-thin slices were made by the LKB NOVA ultramicrotome, incubated with toluidine blue, and observed with the light microscope. Ultra-thin slices with the thickness of 60 nm were obtained and visualized with TEM (Technai10, Philips, Eindhoven, Netherlands, USA).

Quantitative reverse transcription polymerase chain reaction (qRT-PCR) analysis

Total RNA was extracted using TRI-Reagent (Thermo Fisher Scientific, 15596018) and dissolved in 20 μL DEPC-treated water. For mRNA, reverse transcription was obtained with the PrimeScript RT Master Mix (TaKaRa, RR036A) and qRT-PCR was performed with SYBR Premix Ex Taq (Takara, RR820A) on LightCycler 96 system (Roche, Basel, Switzerland). GAPDH was used as negative control. For miRNA, qRT-PCR was conducted with All-in-One miRNA qRT-PCR Detection System 2.0 (Genecopoeia, QP115) and U6 snRNA was the negative control. The gene-specific primers are listed in Table S3.

Protein isolation and western blot analysis

Cells were lysed with IP Lysis buffer (Thermo Fisher Scientific, 87787). After BCA quantification (Thermo Fisher Scientific, A53225), an equal amount of protein was separated by 10% SDS-PAGE and electrophoretically transferred to nitrocellulose membrane (Millipore, Billerica, MA, USA). Proteins were then incubated sequentially with primary antibodies and secondary antibodies. The membrane was visualized with SuperSignal West Pico chemiluminescent substrate (Thermo Fisher Scientific, 34580) on a ChemiDoc imaging system (BioRad, USA). Primary antibodies were as follows: anti-NCX1 (Abcam, ab177952), anti-MYL3 (Abcam, ab108516), anti-MYH6 (Abcam, ab207976), anti-MYH7 (Abcam, ab11083), anti-TPM1 (Abcam, ab109505), anti-TNNC1 (Thermo Fisher Scientific, PA5-116462), anti-Desmoplakin I+II (Abcam, ab71690), anti-Plakoglobin (Abcam, ab184919), anti-N-Cadherin (Abcam, ab18203), anti-Connexin 43 (Thermo Fisher Scientific, 13-8300), anti-cTnT (Thermo Fisher Scientific, MA5-12960), and anti-GAPDH (Abcam, ab181602).

Intracellular calcium transient analysis

NRVMs with or without MWCNTs treatment were loaded with 5 µM fluo-4 AM (Thermo, F14201) and 0.1% Pluronic F-127 solution at 37°C for 30 minutes and washed three times in Tyrode's solution. Fluo-4 AM was excited at 488 nm. Calcium transients were recorded by Nikon microscope (Tokyo, Japan, Ti2-U). Two minutes later, cells were treated with 30 μM KB-R7943 (MCE, HY-15415) and calcium transients were recorded for another two minutes. Fluorescence (F) was normalized to the basal cellular fluorescence (F_0_) to obtain the fluorescence ratio (F/F_0_).

Measurement of myocyte shortening

Myocyte shortening was recorded by Zeiss microscope (Oberkochen, Germany). Cells were maintained on the stage with unchanging the field of view. Two minutes later, cells were treated with 30 μM KB-R7943 (MCE, HY-15415) and myocyte shortening was recorded for another two minutes. The resting and contracted cell length were measured using adobe Photoshop software. Cell shortening was expressed as % resting cell length (RCL), i.e., [(resting cell length – contracted cell length)/ resting cell length]×100.

Recordings of multielectrode array (MEA)

NRVMs/iPSC-CMs were seeded on fibronectin-coated MEA plates and maintained under various experimental conditions. Field potential and beating rate of cardiomyocytes were measured with MEA recording system (Multichannel Systems, Reutlingen, Germany). Data analysis was conducted with the MC_Data Tool (Multichannel Systems, Reutlingen, Germany) and Matlab (MathWorks, Natick, MA, USA).

Sample preparation and tandem mass tag (TMT)-Based quantitative proteomics analysis

Sample preparation and TMT-Based quantitative proteomics analysis were performed by Jingjie PTM Biolabs (Hangzhou) Co. Ltd. (Hangzhou, China). Cell samples were incubated with lysis buffer (8 M urea, 2 mM EDTA, 10 mM DTT, and 1% Protease Inhibitor Cocktail III) and sonicated three times on ice. The remaining debris was removed by centrifugation at 20,000 g at 4°C for 10 min. The protein was precipitated with cold 15% trichloroacetic acid for 2 h at -20°C. After centrifugation at 4°C for 10 min, the supernatant was discarded. The precipitate was washed three times with cold acetone and re-dissolved in the buffer (8 M urea, 100 mM TEAB, pH 8.0). The concentration was measured using a 2-D Quant kit.

For trypsin digestion, about 100 μg protein each sample was digested. Firstly, the protein sample was reduced with 10 mM DTT for 1 h at 37°C and alkylated with 20 mM IAA for 45 minutes at room temperature in the dark. Secondly, to decrease the urea concentration less than 2 M, the protein sample was diluted by adding 100 mM TEAB. Finally, trypsin was added to the at a 1:50 trypsin-to-protein mass ratio for the first digestion overnight and 1:100 trypsin-to-protein mass ratio for a second 4-h digestion. After trypsin digestion, the peptide was desalted on a Strata X C18 SPE column (Phenomenex, Torrance, CA, USA) and then vacuum-dried. Peptides were resolved in 0.5 M TEAB and processed labelling using 6-plex TMT kit following the manufacturer’s protocol. The sample was fractionated by high-pH reverse-phase HPLC using an Agilent 300 Extend C18 column (5 μm particles, 4.6 mm ID, 250 mm length) (Agilent Technologies, Santa Clara, CA, USA) and eluted with the buffer of 10 mM ammonium bicarbonate (pH 10) in acetonitrile. Peptides were first separated with the buffer gradient of 2–60% over 80 min into 80 fractions. Next, the peptides were combined into 18 fractions and dried by vacuum centrifugation. Peptides were then dissolved in 0.1% FA and injected into LC-MS/MS. Peptides were separated by reverse phase ultra-performance liquid chromatography on a C18 analytical column (Acclaim PepMap RSLC, Thermo Fisher Scientific) and analyzed at 400 nL/min flow rate with the following buffer gradient: 7% to 23% solvent B (0.1% FA in 98% ACN) over 26 min, 23% to 35% in 8 min, and 80% in 3 min, which was then held at 80% for the final 3 min. The peptides were detected using Thermo Q ExactiveTM plus tandem mass spectrometry equipped with nanospray ionization source. In the Orbitrap, Intact peptides were detected at a resolution of 70,000 and selected with NCE setting as 30; ion fragments were detected at a resolution of 17,500. A data-dependent procedure alternated between one MS scan and 20 MS/MS scans and then applied for the detection of the top 20 precursor ions above a threshold ion count of 10,000 in the MS survey scan with 30.0 s dynamic exclusion. The applied nanospray voltage was 2.0 kV. Automatic gain control prevented the overfilling of the orbitrap; 5E4 ions were accumulated to generate MS/MS spectra. The m/z scan range was 350–1800 during MS scans and the fixed first mass was set to 100 m/z.

Proteomics data analysis

Proteomics data were processed on the Mascot search engine (v.2.3.0) and searched in the Uniprot Rat database. Trypsin/P was specified as cleavage enzyme allowing up to 2 missing cleavages. Carbamidomethyl on Cys were specified as fixed modification and oxidation on Met was specified as variable modifications. Mass error was set to 10 ppm for precursor ions and 0.02 Da for fragment ions. TMT-6-plex was selected in Mascot for protein quantification. The false discovery rate was adjusted to <1% and peptide ion score was set to ≥20. P < 0.05 and 1.2-fold cutoff are used to define the significantly changed proteins. Enrichment and clustering analysis of different ratio quantiles for the gene ontology (GO) biological process was carried out by the UniProt-GOA database (www. http://www.ebi.ac.uk/GOA/) and InterPro (http://www.ebi.ac.uk/ interpro/) database, converting identified protein ID to UniProt ID and then mapping to GO IDs. The Kyoto Encyclopedia of Genes and Genomes (KEGG) database was used to annotate protein pathway. First, the KEGG online service tools KAAS were applied to annotate the protein’s KEGG database description. Then the results were mapped using KEGG online service tools KEGG mapper.

Dual luciferase reporter analysis

Targeting of NCX1 3′UTR by miR-135a-5p was validated by the dual luciferase reporter analysis in HEK293T cells (ATCC, CRL-3216). HEK293T cells were cultivated in DMEM (Invitrogen, Waltham, MA, USA) containing 10% FBS (Invitrogen, Waltham, MA, USA) at 37°C and 5% CO_2_. The putative binding sequences (wild-type or mutant) in the NCX1 3’UTR were amplified by PCR from rat genomic DNA and cloned into GV272 vector (GeneChem, Shanghai). Dual luciferase reporter gene was co-transfected along with miR-135a-5p plasmid into HEK293T cells in a 24-well plate using Lipofectamine 3000 reagent (Thermo Fisher Scientific, 2292026) following the manufacturer’s protocols with triplicate for each group. Dual luciferase enzyme activities were measured after 48 h of transfection by Dual-Luciferase Reporter 1000 Assay System (Promega, E1980) according to the manufacturer’s protocols.

Statistical analysis

Data are shown as mean ± SEM. The Student’s unpaired t test was applied for statistical analysis by GraphPad Prism (version 9; GraphPad Software, La Jolla, CA) and Microsoft Excel (version 2020; Microsoft Software, Redmond, Washington) and values P < 0.05 was considered significant (*P < 0.05, **P < 0.01, ***P < 0.001 and ****P < 0.0001).

1. Zeevi, G.et al. Automated circuit fabrication and direct characterization of carbon nanotube vibrations. *Nat. Commun.* **7**, 12153 (2016).

2. Zhang, X. et al. Highly selective and active CO_2_ reduction electrocatalysts based on cobalt phthalocyanine/carbon nanotube hybrid structures. *Nat. Commun.* **8**, 14675 (2017).

3. Sun, H. et al. Carbon nanotubes enhance intercalated disc assembly in cardiac myocytes via the β1-integrin-mediated signaling pathway. *Biomaterials* **55**, 84–95 (2015).

4. Martinelli, V. et al. Carbon nanotubes promote growth and spontaneous electrical activity in cultured cardiac myocytes. *Nano Lett.* **12**, 1831–1838 (2012).

**
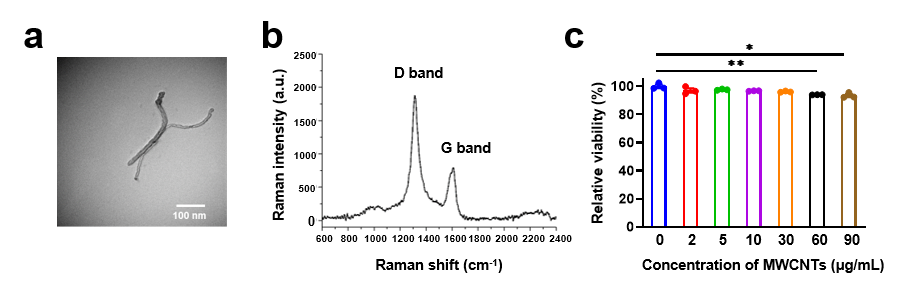
**

**Figure. S1 Characterization and cytotoxicity evaluation of MWCNTs.**

**a,b** Physical characterization of MWCNTs using TEM micrographs (a) and Raman analysis (b). **c** Relative viability of NRVMs after exposure to different concentrations of MWCNTs by CCK-8 assay. n = 3.

**Figure. S2 Intracellular distribution of MWCNTs.**

**a** TEM analysis of NRVMs with MWCNTs treatment for 3 days and 10 days. Z: Z-band, N: Nuclei and MWCNT: multiwalled carbon nanotube. Scale bars, 500 nm. **b** H&E-staining analysis of NRVMs with MWCNTs treatment for 3 days, 7 days and 10 days. Scale bars, 10 µm.


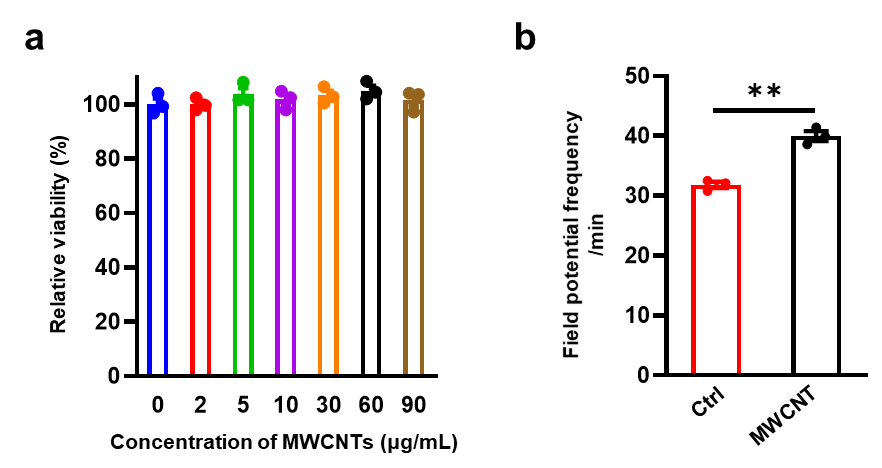


**Figure. S3 Effects of MWCNTs on iPSC-CMs.**

**a** Relative viability of iPSC-CMs after exposure to different concentrations of MWCNTs by CCK-8 assay. **b** MEA assay of iPSC-CMs with 30 μg/mL of MWCNT treatment.

**
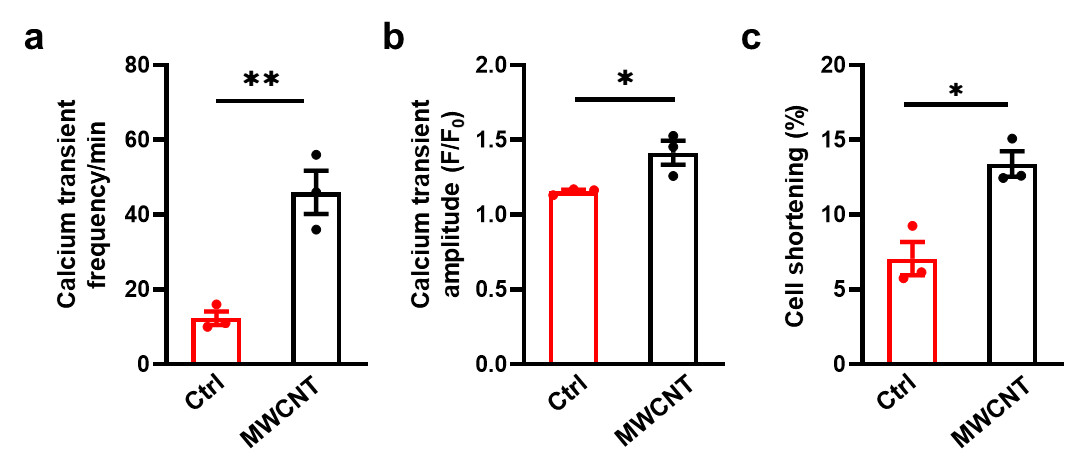
**

**Figure. S4** **Spontaneous calcium transient and cell shortening analysis of NRVMs with MWCNTs treatment.**

**a,b** Statistical evaluation of spontaneous calcium transient of NRVMs with MWCNTs treatment for 10 days. Calcium transient frequency (a): fluctuations per minute. Calcium transient amplitude (b): the maximum of F/F_0_. n =3. **c** Cell shortening of NRVMs with MWCNTs treatment for 10 days. n = 3.

**
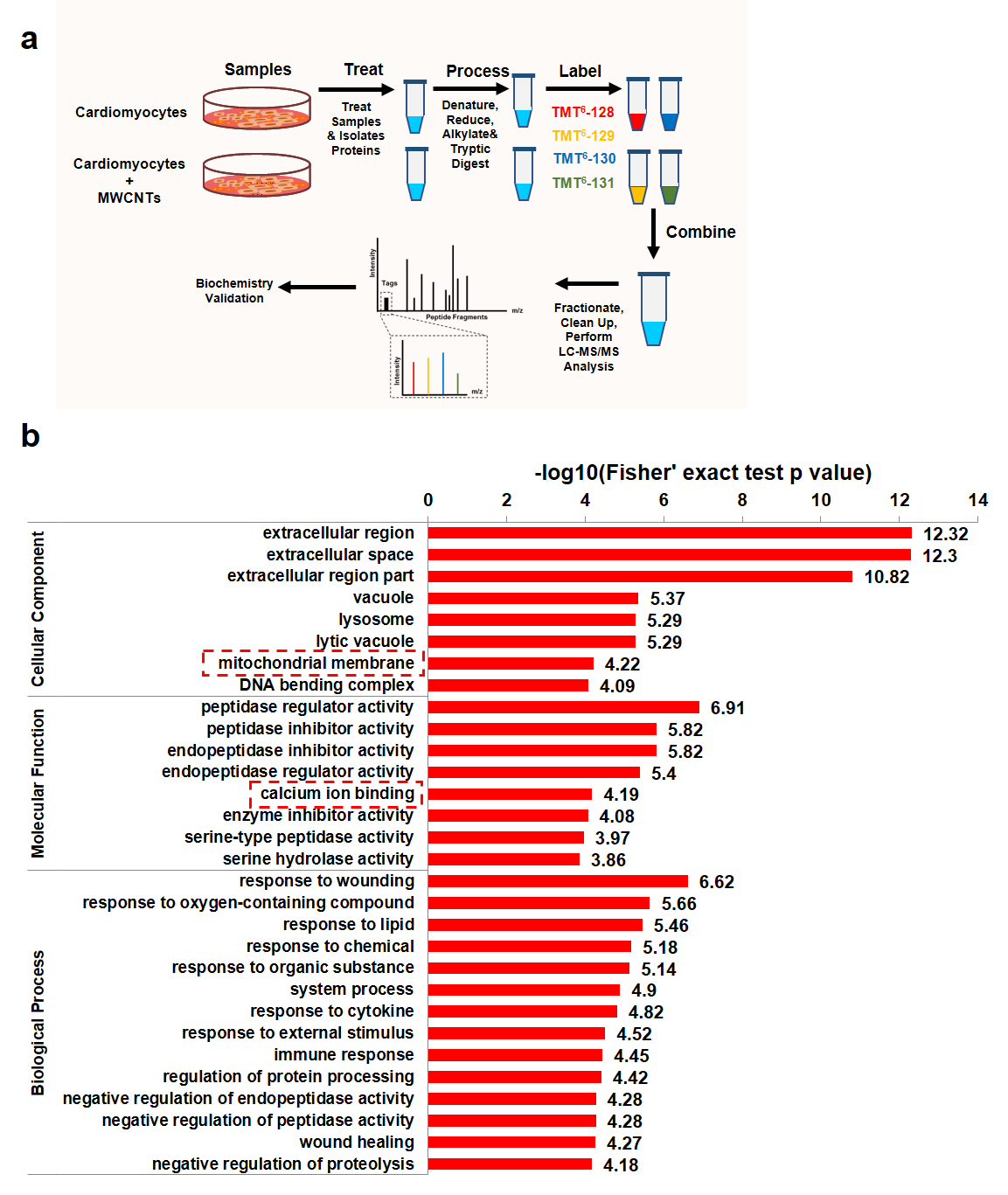
**

**Figure. S5 TMT-Based quantitative proteomics analysis.**

**a** Flowchart of TMT-Based quantitative proteomics analysis in NRVMs with MWCNTs treatment for 10 days. **b** GO analysis of MWCNTs-upregulated proteins in NRVMs.

**
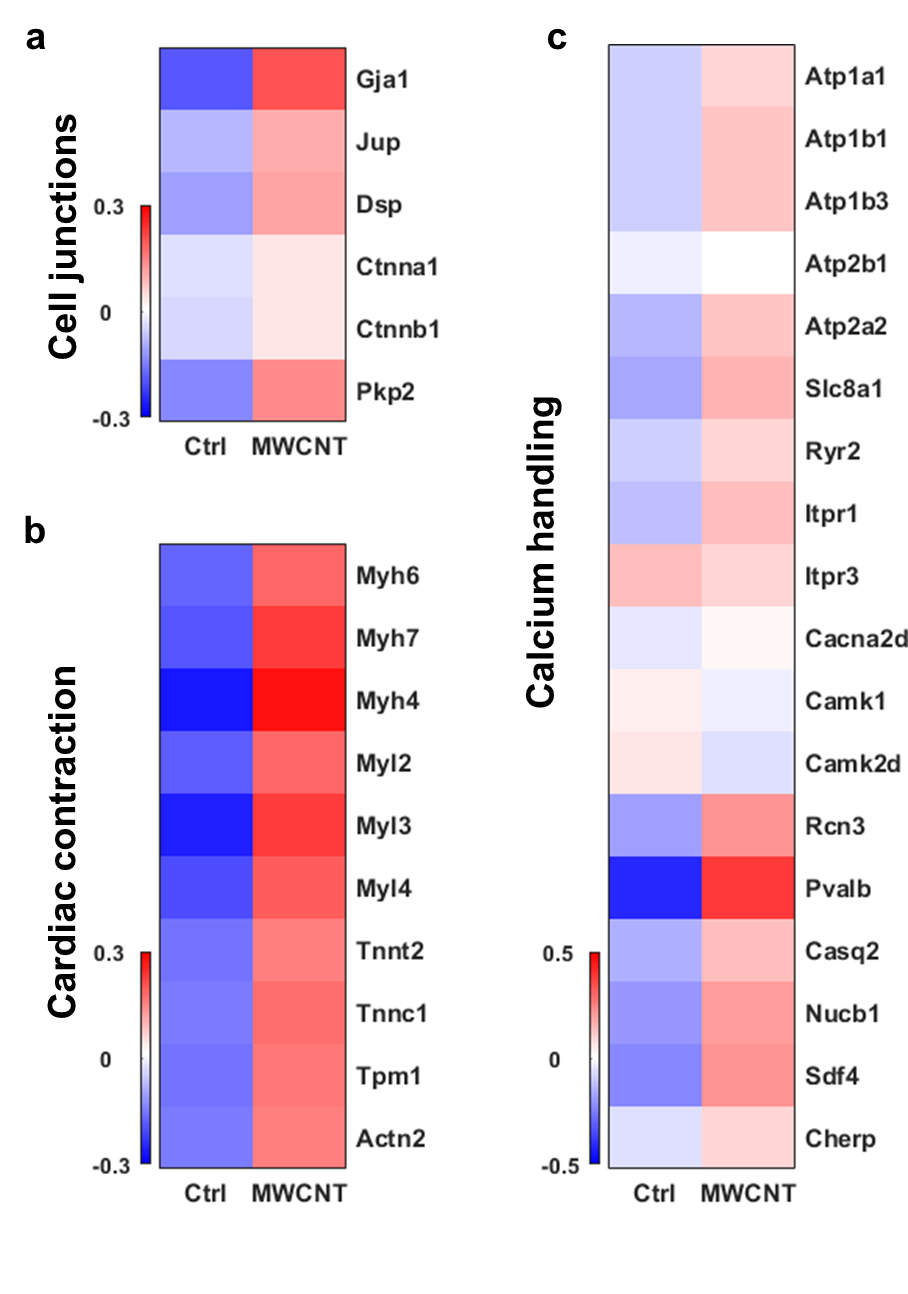
**

**Figure. S6 Heatmap to display different blocks of representative genes in cell junctions (a), cardiac contraction (b) and calcium handling (c). n=3.**

**
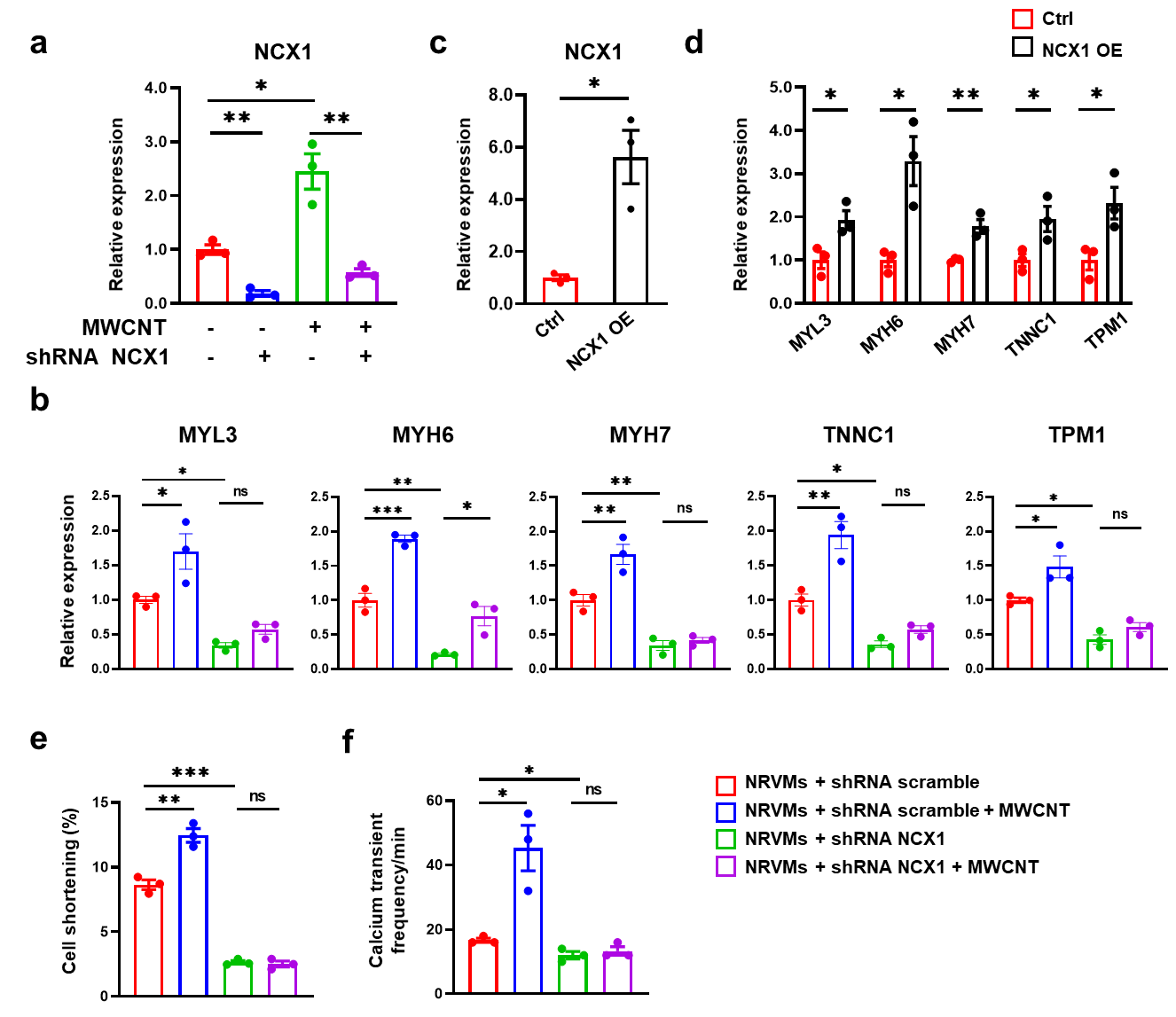
**

**Figure. S7 NCX1 mediated the effects of MWCNTs on cardiac contraction and calcium activity.**

**a** qRT-PCR analysis of NCX1 level in NRVMs with MWCNTs treatment and NCX1 knockdown (KD). n = 3. **b** qRT-PCR analysis of genes in cardiac muscle contraction pathway in NRVMs with NCX1 knockdown and MWCNT treatment. n = 3. **c** qRT-PCR analysis of NCX1 level to evaluate NCX1 overexpression (OE) efficiency in NRVMs. n = 3. **d** qRT-PCR analysis of genes in cardiac muscle contraction pathway in NRVMs after NCX1 overexpression. n = 3. **e,f** Cell shortening (**e**) and spontaneous calcium transient frequency analysis (**f)** of NRVMs with NCX1 knockdown and MWCNT treatment. n = 3.

**Figure. S8 Prediction of miRNAs targeting NCX1.**

**
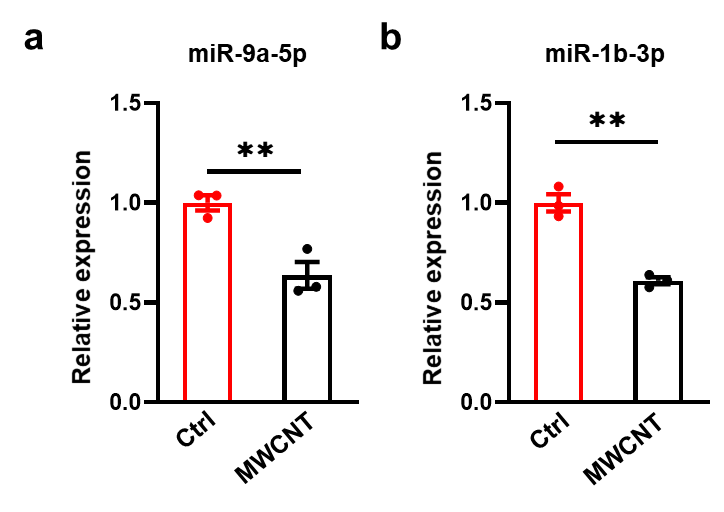
**

**Figure. S9 MiRNAs targeting NCX1 decreased in MWCNTs-treated NRVMs.**

**a, b** qRT-PCR analysis of miR-9a-5p (a) and miR-1b-3p (b) in NRVMs with MWCNTs treatment for 10 days. n = 3.

**
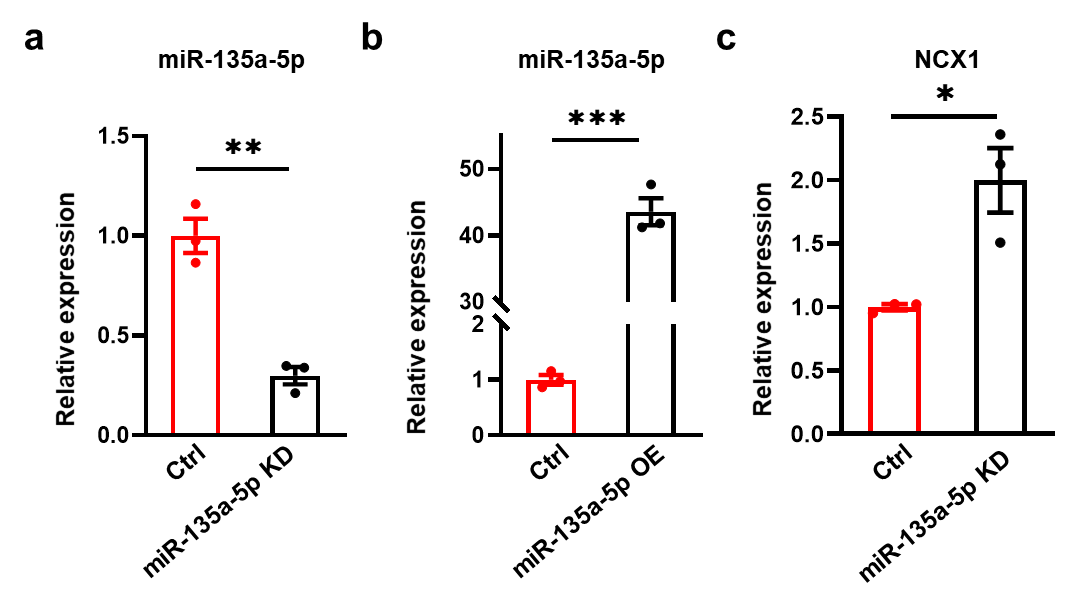
**

**Figure. S10 MiR-135a-5p regulated NCX1 expression level in NRVMs.**

**a, b** qRT-PCR analysis of miR-135a-5p level to evaluate miR-135a-5p knockdown (KD) (a) and overexpression (OE) (b) efficiency in cardiomyocytes. n = 3. **c** qRT-PCR analysis of NCX1 level in cardiomyocytes after miR-135a-5p knockdown. n = 3.


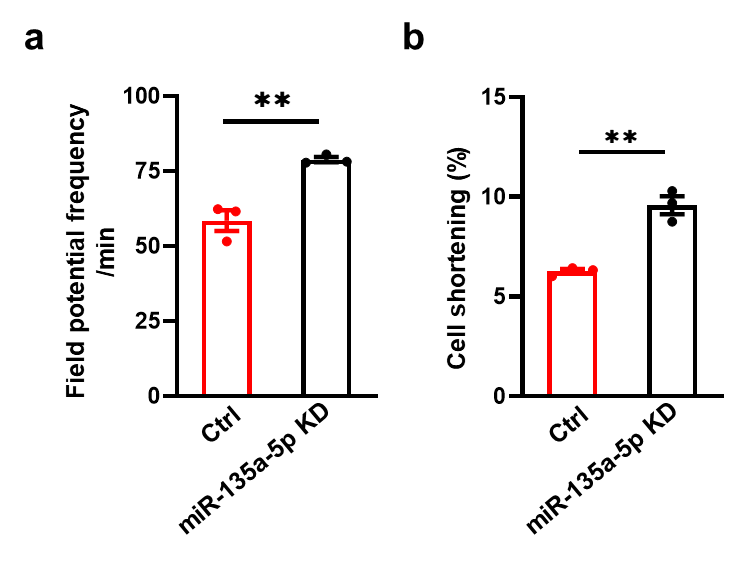


**Figure. S11** **MEA assay (a) and cell shortening assay (b) of NRVMs with miR135a-5p knockdown.** n = 3.

**Table S1. KEGG pathway-based enrichment analysis of upregulated proteins**

| **KEGG pathway** | **Fisher' exact test p value** | **Related proteins** |
| --- | --- | --- |
| rno04142 Lysosome - Rattus norvegicus | 5.05008E-09 | P08081 P45479 Q64194 Q32KJ5 P00786 F7FJQ3 Q641X3 Q6AXR4 P24268 P14562 Q6AYS3 Q6P7A9 P00787 P27615 P17164 P10960 Q6IN37 Q9EQV6 P80067 |
| rno05010 Alzheimer's disease - Rattus norvegicus | 5.39126E-09 | P11951 D4A565 P07861 D4A4P3 P02650 P21571 B2RYW3 P11240 D4A7L4 D4A0T0 A9UMV9 Q68FY0 D3ZF13 Q8CGU6 B2RYS2 F1LPG5 F7EXQ7 P80432 P12075 Q71S46 D3ZLT1 B2RZD6 P31399 Q06000 |
| rno00190 Oxidative phosphorylation - Rattus norvegicus | 1.04928E-08 | P29419 P11661 P11951 D4A565 D4A4P3 P21571 B2RYW3 P11240 D4A7L4 D4A0T0 A9UMV9 Q68FY0 D3ZF13 B2RYS2 F1LPG5 F7EXQ7 P80432 P12075 Q71S46 D3ZLT1 B2RZD6 P31399 |
| rno04260 Cardiac muscle contraction - Rattus norvegicus | 3.69382E-08 | Q4PP99 B2RYS2 P04692 P11951 P02564 P16409 Q01728 P80432 P08733 P02563 P12075 P11240 P17209 P50753 Q68FY0 |
| rno04610 Complement and coagulation cascades - Rattus norvegicus | 1.30378E-07 | Q01177 Q6P734 A0A0G2K7X7 P53813 Q63041 P05371 P08649 P01026 P19637 P06238 |
| rno05012 Parkinson's disease - Rattus norvegicus | 1.37286E-07 | P11661 P11951 D4A565 D4A4P3 P21571 B2RYW3 P11240 D4A7L4 D4A0T0 A9UMV9 Q68FY0 D3ZF13 B2RYS2 F1LPG5 F7EXQ7 P80432 P12075 Q71S46 D3ZLT1 B2RZD6 P31399 |
| rno05016 Huntington's disease - Rattus norvegicus | 1.0074E-06 | P07895 P11951 D4A565 P08081 D4A4P3 P21571 B2RYW3 P11240 D4A7L4 D4A0T0 A9UMV9 Q68FY0 D3ZF13 B2RYS2 F1LPG5 F7EXQ7 P80432 P12075 Q71S46 D3ZLT1 B2RZD6 P31399 |
| rno04932 Non-alcoholic fatty liver disease (NAFLD) - Rattus norvegicus | 4.99601E-06 | P11951 D4A565 D4A4P3 B2RYW3 P11240 D4A7L4 D4A0T0 A9UMV9 Q68FY0 D3ZF13 B2RYS2 F1LPG5 F7EXQ7 P80432 P12075 D3ZLT1 B2RZD6 |
| rno00140 Steroid hormone biosynthesis - Rattus norvegicus | 0.000735009 | Q64678 P08430 P16232 Q63688 P15589 |
| rno05410 Hypertrophic cardiomyopathy (HCM) - Rattus norvegicus | 0.002496596 | Q4PP99 P04692 P02564 P16409 Q01728 P08733 A0A0G2K1V7 P02563 P50753 |
| rno05414 Dilated cardiomyopathy - Rattus norvegicus | 0.003420419 | Q4PP99 P04692 P02564 P16409 Q01728 P08733 A0A0G2K1V7 P02563 P50753 |
| rno05322 Systemic lupus erythematosus - Rattus norvegicus | 0.008501547 | P08649 D3ZCV0 P01026 A0A0G2K7X7 M0RBX6 P62804 |
| rno04261 Adrenergic signaling in cardiomyocytes - Rattus norvegicus | 0.014008821 | Q4PP99 P04692 P02564 P16409 Q01728 P08733 P02563 P50753 P17209 |

**Table S2. KEGG pathway-based enrichment analysis of downregulated proteins**

| **KEGG pathway** | **Fisher' exact test p value** | **Related proteins** |
| --- | --- | --- |
| rno04512 ECM-receptor interaction - Rattus norvegicus | 0.003086271 | F1LNH3 D4A2G6 A0A0G2JV24 D3ZUL3 P04937 |
| rno04510 Focal adhesion - Rattus norvegicus | 0.008009058 | P35465 Q66H76 F1LNH3 D4A2G6 A0A0G2JV24 D3ZUL3 P04937 |
| rno04974 Protein digestion and absorption - Rattus norvegicus | 0.009784963 | F1LNH3 P20909 D3ZUL3 P70560 |

**Table S3. The sequences of primers for mRNA and miRNA detection.**

| **mRNA** | **Forward (5’-3’)** | **Reverse (5’-3’)** |
| --- | --- | --- |
| GAPDH | ACCCATCACCATCTTCCAGGAG | GAAGGGGCGGAGATGATGAC |
| COX6C | GAAGGCTCTCCGTTTCTCCG | ATATGAACCCGCAGACGCTT |
| COX5A | ATGCTCGCTGGGTGACATAC | ATGCCCTCAAAGCAGCATCA |
| COX7C | CCGGATAGCAAGGTCGTGAA | AGGTGGTGAACCTCCGGATA |
| COX5B | GGCTGCATCTGTGAAGAGGA | GTGGACCATTTGGTAGGGCA |
| NCX1 | GATTGGAGAACCCCGTCTGG | TCCTCCTCCTCTTTGCTGGT |
| MYL3 | GCTGAGCCTCTCAGGAAGC | GGAGTGATCTGCGACCATGT |
| MYL4 | GTGCCGACCAGATCGAAGAA | CAAAACTCGCAGCACCTCTG |
| MYH6 | CCGAGTCCCAGGTCAACAAG | TGTGGGATAGCAACAGCGAG |
| TNNC1 | GAGCTGTCGGATCTCTTCCG | TGTCACCGTCCTTCATGAGC |
| cTnT | AAACTCGTGGGAAGGCCAAA | GTGCCTGGCAAGACCTAGAG |
| TPM1 | GGGCTGAGTTTGCAGAGAGA | AGCTCAGAGAGGTGGGACAT |
| Cx43 | ﻿AGCAAGCTAGCGAGCAAAAC | ﻿GAGTTCATGTCCAGCAGCAA |
| N-Cadherin | TGAGTGGAACAGGAACGCTG | CCCTGCATTGGGGTCTATGT |
| plakoglobin | CTGTGGAGAACATCCAGCGT | GTTCCATGAGAGGGGCAGAG |
| desmoplakin | CCTCTCCGGGAGTCCCTC | CTCATGTCTGCGGTCACTCA |
|  |  |  |
| **miRNA** |  |  |
| rno-miR-1b-3p | TGGAATGTAAAGAAGTGTGTATAAA | GTGCAGGGTCCGAGGT |
| rno-miR-135a-5p | GGCTTTTTATTCCTATGTGAA | GTGCAGGGTCCGAGGT |
| rno-miR-9a-5p | TCTTTGGTTATCTAGCTGTATGAAA | GTGCAGGGTCCGAGGT |
| rno-U6 snRNA | CGCTTCGGCAGCACATATACTA | CGCTTCACGAATTTGCGTGTCA |
| rno-pre miR-135a-5p | AAATTCACTCTAGTGCTTTATGGCT | TTCACAATGTATTTCATGGCTTCCA |

**Sequences of lentiviral NCX1 and miR-135a-5p**

**Lentivirus for NCX1 overexpression：**

acgcgtgtagtcttatgcaatactcttgtagtcttgcaacatggtaacgatgagttagcaacatgccttacaaggagagaaaaagcaccgtgcatgccgattggtggaagtaaggtggtacgatcgtgccttattaggaaggcaacagacgggtctgacatggattggacgaaccactgaattgccgcattgcagagatattgtatttaagtgcctagctcgatacaataaacgggtctctctggttagaccagatctgagcctgggagctctctggctaactagggaacccactgcttaagcctcaataaagcttgccttgagtgcttcaagtagtgtgtgcccgtctgttgtgtgactctggtaactagagatccctcagacccttttagtcagtgtggaaaatctctagcagtggcgcccgaacagggacctgaaagcgaaagggaaaccagagctctctcgacgcaggactcggcttgctgaagcgcgcacggcaagaggcgaggggcggcgactggtgagtacgccaaaaattttgactagcggaggctagaaggagagagatgggtgcgagagcgtcagtattaagcgggggagaattagatcgcgatgggaaaaaattcggttaaggccagggggaaagaaaaaatataaattaaaacatatagtatgggcaagcagggagctagaacgattcgcagttaatcctggcctgttagaaacatcagaaggctgtagacaaatactgggacagctacaaccatcccttcagacaggatcagaagaacttagatcattatataatacagtagcaaccctctattgtgtgcatcaaaggatagagataaaagacaccaaggaagctttagacaagatagaggaagagcaaaacaaaagtaagaccaccgcacagcaagcggccactgatcttcagacctggaggaggagatatgagggacaattggagaagtgaattatataaatataaagtagtaaaaattgaaccattaggagtagcacccaccaaggcaaagagaagagtggtgcagagagaaaaaagagcagtgggaataggagctttgttccttgggttcttgggagcagcaggaagcactatgggcgcagcctcaatgacgctgacggtacaggccagacaattattgtctggtatagtgcagcagcagaacaatttgctgagggctattgaggcgcaacagcatctgttgcaactcacagtctggggcatcaagcagctccaggcaagaatcctggctgtggaaagatacctaaaggatcaacagctcctggggatttggggttgctctggaaaactcatttgcaccactgctgtgccttggaatgctagttggagtaataaatctctggaacagattggaatcacacgacctggatggagtgggacagagaaattaacaattacacaagcttaatacactccttaattgaagaatcgcaaaaccagcaagaaaagaatgaacaagaattattggaattagataaatgggcaagtttgtggaattggtttaacataacaaattggctgtggtatataaaattattcataatgatagtaggaggcttggtaggtttaagaatagtttttgctgtactttctatagtgaatagagttaggcagggatattcaccattatcgtttcagacccacctcccaaccccgaggggacccgacaggcccgaaggaatagaagaagaaggtggagagagagacagagacagatccattcgattagtgaacggatctcgacggtatcggttaacttttaaaagaaaaggggggattggggggtacagtgcaggggaaagaatagtagacataatagcaacagacatacaaactaaagaattacaaaaacaaattacaaaattcaaaattttatcgatactagtgacattgattattgactagttattaatagtaatcaattacggggtcattagttcatagcccatatatggagttccgcgttacataacttacggtaaatggcccgcctggctgaccgcccaacgacccccgcccattgacgtcaataatgacgtatgttcccatagtaacgccaatagggactttccattgacgtcaatgggtggagtatttacggtaaactgcccacttggcagtacatcaagtgtatcatatgccaagtacgccccctattgacgtcaatgacggtaaatggcccgcctggcattatgcccagtacatgaccttatgggactttcctacttggcagtacatctacgtattagtcatcgctattaccatggtgatgcggttttggcagtacatcaatgggcgtggatagcggtttgactcacggggatttccaagtctccaccccattgacgtcaatgggagtttgttttggcaccaaaatcaacgggactttccaaaatgtcgtaacaactccgccccattgacgcaaatgggcggtaggcgtgtacggtgggaggtctatataagcagagctccgtttagtgaaccgtcagatcactagaagctttattgcggtagtttatcacagttaaattgctaacgcagtcagtgcttctgacacaacagtctcgaacttaagctgcagtgactctcttaaggtagccttgcagaagttggtcgtgaggcactgggcaggtaagtatcaaggttacaagacaggtttaaggagaccaatagaaactgggcttgtcgagacagagaagactcttgcgtttctgataggcacctattggtcttactgacatccactttgcctttctctccacagggcgcgccgccaccatgcttcgactaagtctcccacccaatgtttcaatgggatttcgtctggtaactctggtggctctcttgtttacccatgttgaccatataactgcagatacagaggcagaaacaggaggaaatgaaaccactgaatgtactggctcatattactgtaagaaaggggtgattctgcccatttgggaaccccaagacccatcttttggggataaaattgctagagcaactgtgtattttgtggccatggtctacatgttcctcggagtttctattattgccgaccggtttatgtcctctatagaagtcatcacctctcaagaaaaggagattaccataaagaaaccaaatggagagaccaccaagactacagtgcgtatctggaatgagactgtgtccaacctgaccttgatggccctgggatcttccgctcctgagattctcctgtctgtcattgaagtgtgtggccataacttcaccgcaggggaccttggtcccagcaccattgtgggaagcgccgccttcaacatgttcatcatcatcgcgctttgtgtttatgtggtcccagatggagagacgaggaagattaaacatctgcgtgtgttctttgtgacagcagcctggagcatctttgcctatacctggctttacataattttgtctgtcagctctcctggtgtcgtggaggtctgggaaggattactcactttcttcttctttcccatctgtgttgtgttcgcttgggttgcagacaggcggcttctcttttacaagtatgtctacaagcggtacagggctggcaagcagagggggatgatcattgaacatgaaggagacagaccagcttccaaaactgaaattgaaatggatgggaaagtagtcaactcccacgttgacaatttcttagatggcgctctggttttggaagtcgatgagagggaccaagatgacgaggaagccaggcgtgagatggcaaggattctgaaggaacttaagcagaagcatcccgacaaagagatcgaacaattaatagaattagccaactatcaagtcctaagtcagcagcaaaagagccgagcattttaccgaattcaagctactcgcctgatgactggagctggtaacattttgaagaggcatgcagctgaccaagcgaggaaggctgtcagcatgcatgaagtcaacatggatgtggttgaaaatgacccagtcagtaaggtcttctttgagcaagggacataccagtgtctagaaaactgtggtactgtggccctcaccattattcgaagagggggtgacttgaccaacactgtgtttgttgacttcaggacggaagatggcacagccaatgctgggtctgattatgagttcacggaagggactgtgatcttcaaacctggggagacccagaaggaaatcagagttggcatcattgatgatgatatctttgaagaagatgaaaactttcttgtgcatcttagcaacgtcagggtctcttcagaagtctcggaagatggcatactagactccaatcacgtgtctgcgattgcttgtctcgggtcacccaacactgccaccataaccatttttgatgatgaccacgcgggcatctttactttcgaggaacccgtgactcacgtgagcgagagcattggcatcatggaggtgaaggtgctgagaacctctggagcgcgaggaaatgttatcattccctataaaaccattgaaggcacagcccgaggtggaggggaggactttgaggacacctgtggagagctggaattccagaatgatgaaatagtcaaaacaatatcagtcaaggtaatcgatgacgaggagtatgagaaaaacaagaccttcttcattgagattggagaaccccgtctggtggagatgagtgagaagaaaggtggcttcacattaacagaggagtacgatgacaagcagccactgaccagcaaagaggaggaggagaggcgcattgcagaaatggggcgccccattctaggcgaacacaccaagctggaagtgatcattgaagagtcttacgaattcaagagcactgtggacaaactcattaagaagacgaacctggccctcgtggtggggaccaacagctggagagagcagttcattgaagcgatcaccgtcagcgctggggaagatgacgatgatgatgaatgtggggaggagaagctgccctcctgttttgattacgtgatgcactttctcacagtgttctggaaggttctgtttgccttcgtcccacctacagaatattggaatggctgggcctgcttcattgtctccatcctcatgatcggcctactgacagccttcattggagatctggcttcccactttggctgcaccattggtctgaaagattccgtgactgcagttgtgtttgtcgctcttggaacctcagtgccagacacatttgccagcaaagtagcagctacccaggaccagtatgcagatgcgtccataggcaatgtcaccggaagcaacgctgtgaatgtcttcctgggaatcggcgtggcctggtccattgctgccatctaccatgcggccaacggggaacagttcaaagtgtcccctggcacgctagctttctctgtcactctcttcaccatttttgctttcatcaacgtgggggtgctgctgtatcggcggaggccagaaataggaggtgagctgggtgggccccggactgccaagctcctcacatcttccctgtttgtgctcctgtggctcttgtacattttcttctcctccctggaggcctactgccacataaaaggcttccctaggggaagcggagccacgaatttctcgctactcaagcaggccggtgatgtcgaggaaaaccctggtcctatggtgtctagaagcaagggcgaggagctgttcaccggggtggtgcccatcctggtcgagctggacggcgacgtaaacggccacaagttcagcgtgtccggcgagggcgagggcgatgccacctacggcaagctgaccctgaagttcatctgcaccaccggcaagctgcccgtgccctggcccaccctcgtgaccaccctgacctacggcgtgcagtgcttcagccgctaccccgaccacatgaagcagcacgacttcttcaagtccgccatgcccgaaggctacgtccaggagcgcaccatcttcttcaaggacgacggcaactacaagacccgcgccgaggtgaagttcgagggcgacaccctggtgaaccgcatcgagctgaagggcatcgacttcaaggaggacggcaacatcctggggcacaagctggagtacaactacaacagccacaacgtctatatcatggccgacaagcagaagaacggcatcaaggtgaacttcaagatccgccacaacatcgaggacggcagcgtgcagctcgccgaccactaccagcagaacacccccatcggcgacggccccgtgctgctgcccgacaaccactacctgagcacccagtccgccctgagcaaagaccccaacgagaagcgcgatcacatggtcctgctggagttcgtgaccgccgccgggatcactctcggcatggacgagctgtacaagtaagtcgacaatcaacctctggattacaaaatttgtgaaagattgactggtattcttaactatgttgctccttttacgctatgtggatacgctgctttaatgcctttgtatcatgctattgcttcccgtatggctttcattttctcctccttgtataaatcctggttgctgtctctttatgaggagttgtggcccgttgtcaggcaacgtggcgtggtgtgcactgtgtttgctgacgcaacccccactggttggggcattgccaccacctgtcagctcctttccgggactttcgctttccccctccctattgccacggcggaactcatcgccgcctgccttgcccgctgctggacaggggctcggctgttgggcactgacaattccgtggtgttgtcggggaaatcatcgtcctttccttggctgctcgcctgtgttgccacctggattctgcgcgggacgtccttctgctacgtcccttcggccctcaatccagcggaccttccttcccgcggcctgctgccggctctgcggcctcttccgcgtcttcgccttcgccctcagacgagtcggatctccctttgggccgcctccccgcctggtacctttaagaccaatgacttacaaggcagctgtagatcttagccactttttaaaagaaaaggggggactggaagggctaattcactcccaacgaaaataagatctgctttttgcttgtactgggtctctctggttagaccagatctgagcctgggagctctctggctaactagggaacccactgcttaagcctcaataaagcttgccttgagtgcttcaagtagtgtgtgcccgtctgttgtgtgactctggtaactagagatccctcagacccttttagtcagtgtggaaaatctctagcagtagtagttcatgtcatcttattattcagtatttataacttgcaaagaaatgaatatcagagagtgagaggaacttgtttattgcagcttataatggttacaaataaagcaatagcatcacaaatttcacaaataaagcatttttttcactgcattctagttgtggtttgtccaaactcatcaatgtatcttatcatgtctggctctagctatcccgcccctaactccgcccagttccgcccattctccgccccatggctgactaattttttttatttatgcagaggccgaggccgcctcggcctctgagctattccagaagtagtgaggaggcttttttggaggcctagacttttgcagagacggcccaaattcgtaatcatggtcatagctgtttcctgtgtgaaattgttatccgctcacaattccacacaacatacgagccggaagcataaagtgtaaagcctggggtgcctaatgagtgagctaactcacattaattgcgttgcgctcactgcccgctttccagtcgggaaacctgtcgtgccagctgcattaatgaatcggccaacgcgcggggagaggcggtttgcgtattgggcgctcttccgcttcctcgctcactgactcgctgcgctcggtcgttcggctgcggcgagcggtatcagctcactcaaaggcggtaatacggttatccacagaatcaggggataacgcaggaaagaacatgtgagcaaaaggccagcaaaaggccaggaaccgtaaaaaggccgcgttgctggcgtttttccataggctccgcccccctgacgagcatcacaaaaatcgacgctcaagtcagaggtggcgaaacccgacaggactataaagataccaggcgtttccccctggaagctccctcgtgcgctctcctgttccgaccctgccgcttaccggatacctgtccgcctttctcccttcgggaagcgtggcgctttctcatagctcacgctgtaggtatctcagttcggtgtaggtcgttcgctccaagctgggctgtgtgcacgaaccccccgttcagcccgaccgctgcgccttatccggtaactatcgtcttgagtccaacccggtaagacacgacttatcgccactggcagcagccactggtaacaggattagcagagcgaggtatgtaggcggtgctacagagttcttgaagtggtggcctaactacggctacactagaaggacagtatttggtatctgcgctctgctgaagccagttaccttcggaaaaagagttggtagctcttgatccggcaaacaaaccaccgctggtagcggtggtttttttgtttgcaagcagcagattacgcgcagaaaaaaaggatctcaagaagatcctttgatcttttctacggggtctgacgctcagtggaacgaaaactcacgttaagggattttggtcatgagattatcaaaaaggatcttcacctagatccttttaaattaaaaatgaagttttaaatcaatctaaagtatatatgagtaaacttggtctgacagttaccaatgcttaatcagtgaggcacctatctcagcgatctgtctatttcgttcatccatagttgcctgactccccgtcgtgtagataactacgatacgggagggcttaccatctggccccagtgctgcaatgataccgcgagacccacgctcaccggctccagatttatcagcaataaaccagccagccggaagggccgagcgcagaagtggtcctgcaactttatccgcctccatccagtctattaattgttgccgggaagctagagtaagtagttcgccagttaatagtttgcgcaacgttgttgccattgctacaggcatcgtggtgtcacgctcgtcgtttggtatggcttcattcagctccggttcccaacgatcaaggcgagttacatgatcccccatgttgtgcaaaaaagcggttagctccttcggtcctccgatcgttgtcagaagtaagttggccgcagtgttatcactcatggttatggcagcactgcataattctcttactgtcatgccatccgtaagatgcttttctgtgactggtgagtactcaaccaagtcattctgagaatagtgtatgcggcgaccgagttgctcttgcccggcgtcaatacgggataataccgcgccacatagcagaactttaaaagtgctcatcattggaaaacgttcttcggggcgaaaactctcaaggatcttaccgctgttgagatccagttcgatgtaacccactcgtgcacccaactgatcttcagcatcttttactttcaccagcgtttctgggtgagcaaaaacaggaaggcaaaatgccgcaaaaaagggaataagggcgacacggaaatgttgaatactcatactcttcctttttcaatattattgaagcatttatcagggttattgtctcatgagcggatacatatttgaatgtatttagaaaaataaacaaataggggttccgcgcacatttccccgaaaagtgccacctgacgtctaagaaaccattattatcatgacattaacctataaaaataggcgtatcacgaggccctttcgtctcgcgcgtttcggtgatgacggtgaaaacctctgacacatgcagctcccggagacggtcacagcttgtctgtaagcggatgccgggagcagacaagcccgtcagggcgcgtcagcgggtgttggcgggtgtcggggctggcttaactatgcggcatcagagcagattgtactgagagtgcaccatatgcggtgtgaaataccgcacagatgcgtaaggagaaaataccgcatcaggcgccattcgccattcaggctgcgcaactgttgggaagggcgatcggtgcgggcctcttcgctattacgccagctggcgaaagggggatgtgctgcaaggcgattaagttgggtaacgccagggttttcccagtcacgacgttgtaaaacgacggccagtgccaagctg

**Lentivirus for NCX1 knockdown：**

acgcgtgtagtcttatgcaatactcttgtagtcttgcaacatggtaacgatgagttagcaacatgccttacaaggagagaaaaagcaccgtgcatgccgattggtggaagtaaggtggtacgatcgtgccttattaggaaggcaacagacgggtctgacatggattggacgaaccactgaattgccgcattgcagagatattgtatttaagtgcctagctcgatacaataaacgggtctctctggttagaccagatctgagcctgggagctctctggctaactagggaacccactgcttaagcctcaataaagcttgccttgagtgcttcaagtagtgtgtgcccgtctgttgtgtgactctggtaactagagatccctcagacccttttagtcagtgtggaaaatctctagcagtggcgcccgaacagggacctgaaagcgaaagggaaaccagagctctctcgacgcaggactcggcttgctgaagcgcgcacggcaagaggcgaggggcggcgactggtgagtacgccaaaaattttgactagcggaggctagaaggagagagatgggtgcgagagcgtcagtattaagcgggggagaattagatcgcgatgggaaaaaattcggttaaggccagggggaaagaaaaaatataaattaaaacatatagtatgggcaagcagggagctagaacgattcgcagttaatcctggcctgttagaaacatcagaaggctgtagacaaatactgggacagctacaaccatcccttcagacaggatcagaagaacttagatcattatataatacagtagcaaccctctattgtgtgcatcaaaggatagagataaaagacaccaaggaagctttagacaagatagaggaagagcaaaacaaaagtaagaccaccgcacagcaagcggccactgatcttcagacctggaggaggagatatgagggacaattggagaagtgaattatataaatataaagtagtaaaaattgaaccattaggagtagcacccaccaaggcaaagagaagagtggtgcagagagaaaaaagagcagtgggaataggagctttgttccttgggttcttgggagcagcaggaagcactatgggcgcagcctcaatgacgctgacggtacaggccagacaattattgtctggtatagtgcagcagcagaacaatttgctgagggctattgaggcgcaacagcatctgttgcaactcacagtctggggcatcaagcagctccaggcaagaatcctggctgtggaaagatacctaaaggatcaacagctcctggggatttggggttgctctggaaaactcatttgcaccactgctgtgccttggaatgctagttggagtaataaatctctggaacagattggaatcacacgacctggatggagtgggacagagaaattaacaattacacaagcttaatacactccttaattgaagaatcgcaaaaccagcaagaaaagaatgaacaagaattattggaattagataaatgggcaagtttgtggaattggtttaacataacaaattggctgtggtatataaaattattcataatgatagtaggaggcttggtaggtttaagaatagtttttgctgtactttctatagtgaatagagttaggcagggatattcaccattatcgtttcagacccacctcccaaccccgaggggacccgacaggcccgaaggaatagaagaagaaggtggagagagagacagagacagatccattcgattagtgaacggatctcgacggtatcggttaacttttaaaagaaaaggggggattggggggtacagtgcaggggaaagaatagtagacataatagcaacagacatacaaactaaagaattacaaaaacaaattacaaaattcaaaattttatcgatactagtgagggcctatttcccatgattccttcatatttgcatatacgatacaaggctgttagagagataattggaattaatttgactgtaaacacaaagatattagtacaaaatacgtgacgtagaaagtaataatttcttgggtagtttgcagttttaaaattatgttttaaaatggactatcatatgcttaccgtaacttgaaagtatttcgatttcttggctttatatatcttgtggaaaggacgaaacaccgggcgtgagatggcaaggattctttcaagagaagaatccttgccatctcacgctttttaattcacataacttacggtaaatggcccgcctggctgaccgcccaacgacccccgcccattgacgtcaataatgacgtatgttcccatagtaacgccaatagggactttccattgacgtcaatgggtggagtatttacggtaaactgcccacttggcagtacatcaagtgtatcatatgccaagtacgccccctattgacgtcaatgacggtaaatggcccgcctggcattatgcccagtacatgaccttatgggactttcctacttggcagtacatctacgtattagtcatcgctattaccatggtgatgcggttttggcagtacatcaatgggcgtggatagcggtttgactcacggggatttccaagtctccaccccattgacgtcaatgggagtttgttttggcaccaaaatcaacgggactttccaaaatgtcgtaacaactccgccccattgacgcaaatgggcggtaggcgtgtacggtgggaggtctatataagcagagctggtttagtgaaccgtcagatccgctagcgccaccatggtgagcaagggcgaggagctgttcaccggggtggtgcccatcctggtcgagctggacggcgacgtaaacggccacaagttcagcgtgtccggcgagggcgagggcgatgccacctacggcaagctgaccctgaagttcatctgcaccaccggcaagctgcccgtgccctggcccaccctcgtgaccaccctgacctacggcgtgcagtgcttcagccgctaccccgaccacatgaagcagcacgacttcttcaagtccgccatgcccgaaggctacgtccaggagcgcaccatcttcttcaaggacgacggcaactacaagacccgcgccgaggtgaagttcgagggcgacaccctggtgaaccgcatcgagctgaagggcatcgacttcaaggaggacggcaacatcctggggcacaagctggagtacaactacaacagccacaacgtctatatcatggccgacaagcagaagaacggcatcaaggtgaacttcaagatccgccacaacatcgaggacggcagcgtgcagctcgccgaccactaccagcagaacacccccatcggcgacggccccgtgctgctgcccgacaaccactacctgagcacccagtccgccctgagcaaagaccccaacgagaagcgcgatcacatggtcctgctggagttcgtgaccgccgccgggatcactctcggcatggacgagctgtacaagtgagtcgacaatcaacctctggattacaaaatttgtgaaagattgactggtattcttaactatgttgctccttttacgctatgtggatacgctgctttaatgcctttgtatcatgctattgcttcccgtatggctttcattttctcctccttgtataaatcctggttgctgtctctttatgaggagttgtggcccgttgtcaggcaacgtggcgtggtgtgcactgtgtttgctgacgcaacccccactggttggggcattgccaccacctgtcagctcctttccgggactttcgctttccccctccctattgccacggcggaactcatcgccgcctgccttgcccgctgctggacaggggctcggctgttgggcactgacaattccgtggtgttgtcggggaaatcatcgtcctttccttggctgctcgcctgtgttgccacctggattctgcgcgggacgtccttctgctacgtcccttcggccctcaatccagcggaccttccttcccgcggcctgctgccggctctgcggcctcttccgcgacttcgccttcgccctcagacgagtcggatctccctttgggccgcctccccgcctggtacctttaagaccaatgacttacaaggcagctgtagatcttagccactttttaaaagaaaaggggggactggaagggctaattcactcccaacgaaaataagatctgctttttgcttgtactgggtctctctggttagaccagatctgagcctgggagctctctggctaactagggaacccactgcttaagcctcaataaagcttgccttgagtgcttcaagtagtgtgtgcccgtctgttgtgtgactctggtaactagagatccctcagacccttttagtcagtgtggaaaatctctagcagtagtagttcatgtcatcttattattcagtatttataacttgcaaagaaatgaatatcagagagtgagaggaacttgtttattgcagcttataatggttacaaataaagcaatagcatcacaaatttcacaaataaagcatttttttcactgcattctagttgtggtttgtccaaactcatcaatgtatcttatcatgtctggctctagctatcccgcccctaactccgcccagttccgcccattctccgccccatggctgactaattttttttatttatgcagaggccgaggccgcctcggcctctgagctattccagaagtagtgaggaggcttttttggaggcctagacttttgcagagacggcccaaattcgtaatcatggtcatagctgtttcctgtgtgaaattgttatccgctcacaattccacacaacatacgagccggaagcataaagtgtaaagcctggggtgcctaatgagtgagctaactcacattaattgcgttgcgctcactgcccgctttccagtcgggaaacctgtcgtgccagctgcattaatgaatcggccaacgcgcggggagaggcggtttgcgtattgggcgctcttccgcttcctcgctcactgactcgctgcgctcggtcgttcggctgcggcgagcggtatcagctcactcaaaggcggtaatacggttatccacagaatcaggggataacgcaggaaagaacatgtgagcaaaaggccagcaaaaggccaggaaccgtaaaaaggccgcgttgctggcgtttttccataggctccgcccccctgacgagcatcacaaaaatcgacgctcaagtcagaggtggcgaaacccgacaggactataaagataccaggcgtttccccctggaagctccctcgtgcgctctcctgttccgaccctgccgcttaccggatacctgtccgcctttctcccttcgggaagcgtggcgctttctcatagctcacgctgtaggtatctcagttcggtgtaggtcgttcgctccaagctgggctgtgtgcacgaaccccccgttcagcccgaccgctgcgccttatccggtaactatcgtcttgagtccaacccggtaagacacgacttatcgccactggcagcagccactggtaacaggattagcagagcgaggtatgtaggcggtgctacagagttcttgaagtggtggcctaactacggctacactagaaggacagtatttggtatctgcgctctgctgaagccagttaccttcggaaaaagagttggtagctcttgatccggcaaacaaaccaccgctggtagcggtggtttttttgtttgcaagcagcagattacgcgcagaaaaaaaggatctcaagaagatcctttgatcttttctacggggtctgacgctcagtggaacgaaaactcacgttaagggattttggtcatgagattatcaaaaaggatcttcacctagatccttttaaattaaaaatgaagttttaaatcaatctaaagtatatatgagtaaacttggtctgacagttaccaatgcttaatcagtgaggcacctatctcagcgatctgtctatttcgttcatccatagttgcctgactccccgtcgtgtagataactacgatacgggagggcttaccatctggccccagtgctgcaatgataccgcgagacccacgctcaccggctccagatttatcagcaataaaccagccagccggaagggccgagcgcagaagtggtcctgcaactttatccgcctccatccagtctattaattgttgccgggaagctagagtaagtagttcgccagttaatagtttgcgcaacgttgttgccattgctacaggcatcgtggtgtcacgctcgtcgtttggtatggcttcattcagctccggttcccaacgatcaaggcgagttacatgatcccccatgttgtgcaaaaaagcggttagctccttcggtcctccgatcgttgtcagaagtaagttggccgcagtgttatcactcatggttatggcagcactgcataattctcttactgtcatgccatccgtaagatgcttttctgtgactggtgagtactcaaccaagtcattctgagaatagtgtatgcggcgaccgagttgctcttgcccggcgtcaatacgggataataccgcgccacatagcagaactttaaaagtgctcatcattggaaaacgttcttcggggcgaaaactctcaaggatcttaccgctgttgagatccagttcgatgtaacccactcgtgcacccaactgatcttcagcatcttttactttcaccagcgtttctgggtgagcaaaaacaggaaggcaaaatgccgcaaaaaagggaataagggcgacacggaaatgttgaatactcatactcttcctttttcaatattattgaagcatttatcagggttattgtctcatgagcggatacatatttgaatgtatttagaaaaataaacaaataggggttccgcgcacatttccccgaaaagtgccacctgacgtctaagaaaccattattatcatgacattaacctataaaaataggcgtatcacgaggccctttcgtctcgcgcgtttcggtgatgacggtgaaaacctctgacacatgcagctcccggagacggtcacagcttgtctgtaagcggatgccgggagcagacaagcccgtcagggcgcgtcagcgggtgttggcgggtgtcggggctggcttaactatgcggcatcagagcagattgtactgagagtgcaccatatgcggtgtgaaataccgcacagatgcgtaaggagaaaataccgcatcaggcgccattcgccattcaggctgcgcaactgttgggaagggcgatcggtgcgggcctcttcgctattacgccagctggcgaaagggggatgtgctgcaaggcgattaagttgggtaacgccagggttttcccagtcacgacgttgtaaaacgacggccagtgccaagctg

**Lentivirus for miR-135a-5p overexpression：**

acgcgtgtagtcttatgcaatactcttgtagtcttgcaacatggtaacgatgagttagcaacatgccttacaaggagagaaaaagcaccgtgcatgccgattggtggaagtaaggtggtacgatcgtgccttattaggaaggcaacagacgggtctgacatggattggacgaaccactgaattgccgcattgcagagatattgtatttaagtgcctagctcgatacaataaacgggtctctctggttagaccagatctgagcctgggagctctctggctaactagggaacccactgcttaagcctcaataaagcttgccttgagtgcttcaagtagtgtgtgcccgtctgttgtgtgactctggtaactagagatccctcagacccttttagtcagtgtggaaaatctctagcagtggcgcccgaacagggacctgaaagcgaaagggaaaccagagctctctcgacgcaggactcggcttgctgaagcgcgcacggcaagaggcgaggggcggcgactggtgagtacgccaaaaattttgactagcggaggctagaaggagagagatgggtgcgagagcgtcagtattaagcgggggagaattagatcgcgatgggaaaaaattcggttaaggccagggggaaagaaaaaatataaattaaaacatatagtatgggcaagcagggagctagaacgattcgcagttaatcctggcctgttagaaacatcagaaggctgtagacaaatactgggacagctacaaccatcccttcagacaggatcagaagaacttagatcattatataatacagtagcaaccctctattgtgtgcatcaaaggatagagataaaagacaccaaggaagctttagacaagatagaggaagagcaaaacaaaagtaagaccaccgcacagcaagcggccactgatcttcagacctggaggaggagatatgagggacaattggagaagtgaattatataaatataaagtagtaaaaattgaaccattaggagtagcacccaccaaggcaaagagaagagtggtgcagagagaaaaaagagcagtgggaataggagctttgttccttgggttcttgggagcagcaggaagcactatgggcgcagcctcaatgacgctgacggtacaggccagacaattattgtctggtatagtgcagcagcagaacaatttgctgagggctattgaggcgcaacagcatctgttgcaactcacagtctggggcatcaagcagctccaggcaagaatcctggctgtggaaagatacctaaaggatcaacagctcctggggatttggggttgctctggaaaactcatttgcaccactgctgtgccttggaatgctagttggagtaataaatctctggaacagattggaatcacacgacctggatggagtgggacagagaaattaacaattacacaagcttaatacactccttaattgaagaatcgcaaaaccagcaagaaaagaatgaacaagaattattggaattagataaatgggcaagtttgtggaattggtttaacataacaaattggctgtggtatataaaattattcataatgatagtaggaggcttggtaggtttaagaatagtttttgctgtactttctatagtgaatagagttaggcagggatattcaccattatcgtttcagacccacctcccaaccccgaggggacccgacaggcccgaaggaatagaagaagaaggtggagagagagacagagacagatccattcgattagtgaacggatctcgacggtatcggttaacttttaaaagaaaaggggggattggggggtacagtgcaggggaaagaatagtagacataatagcaacagacatacaaactaaagaattacaaaaacaaattacaaaattcaaaattttatcgatactagtgacattgattattgactagttattaatagtaatcaattacggggtcattagttcatagcccatatatggagttccgcgttacataacttacggtaaatggcccgcctggctgaccgcccaacgacccccgcccattgacgtcaataatgacgtatgttcccatagtaacgccaatagggactttccattgacgtcaatgggtggagtatttacggtaaactgcccacttggcagtacatcaagtgtatcatatgccaagtacgccccctattgacgtcaatgacggtaaatggcccgcctggcattatgcccagtacatgaccttatgggactttcctacttggcagtacatctacgtattagtcatcgctattaccatggtgatgcggttttggcagtacatcaatgggcgtggatagcggtttgactcacggggatttccaagtctccaccccattgacgtcaatgggagtttgttttggcaccaaaatcaacgggactttccaaaatgtcgtaacaactccgccccattgacgcaaatgggcggtaggcgtgtacggtgggaggtctatataagcagagctccgtttagtgaaccgtcagatcactagaagctttattgcggtagtttatcacagttaaattgctaacgcagtcagtgcttctgacacaacagtctcgaacttaagctgcagtgactctcttaaggtagccttgcagaagttggtcgtgaggcactgggcaggtaagtatcaaggttacaagacaggtttaaggagaccaatagaaactgggcttgtcgagacagagaagactcttgcgtttctgataggcacctattggtcttactgacatccactttgcctttctctccacagggcgcgccttccaagttcaatgtccaagtgatgagcttttctccaggaattctccccagtgtcctttgtttctggtgacattctgtggttgaatgttaccgagtgtctcctggtcttgtttctggtcctcgtagcgaagaatgtcattgaaggataaaaatcttgttaatttgtgatgttccaatctaagtgtgcttcgtgtcccttacgtgttagcctaaaatgttctttaatgtagcatgaaatgattggttggcttggaaatggttttgaagtcgtgtgaagaaaataagttttacatccggccaagataaattcactctagtgctttatggctttttattcctatgtgatcgtaataaagtctcatgtagggatggaagccatgaaatacattgtgaaaattcatcaactaagaaggggccatcagaatagagaactagagcctctggaacgtgctggagacgtgagctagtggacagctctgctaccctctttggtgttccttgagtgtcttcatccttaggcattgacagctaaggaatggaggttgcaagagttctgtcatcttcctgaccaccgattaacatgcacatttgactttgcgcatttctgtctatcatacattttttcccctccgtttttctactaatgatcatgctctggaagaatggttaatagcccactcacttgtaggcagcattccaaggatccctgtgccttctagttgccagccatctgttgtttgcccctcccccgtgccttccttgaccctggaaggtgccactcccactgtcctttcctaataaaatgaggaaattgcatcgcattgtctgagtaggtgtcattctattctggggggtggggtggggcaggacagcaagggggaggattgggaagagaatagcaggcatgctggggacctagggggcagagcgcacatcgcccacagtccccgagaagttggggggaggggtcggcaattgatccggtgcctagagaaggtggcgcggggtaaactgggaaagtgatgtcgtgtactggctccgcctttttcccgagggtgggggagaaccgtatataagtgcagtagtcgccgtgaacgttctttttcgcaacgggtttgccgccagaacacaggctagcatggtgagcaagggcgaggagctgttcaccggggtggtgcccatcctggtcgagctggacggcgacgtaaacggccacaagttcagcgtgtccggcgagggcgagggcgatgccacctacggcaagctgaccctgaagttcatctgcaccaccggcaagctgcccgtgccctggcccaccctcgtgaccaccctgacctacggcgtgcagtgcttcagccgctaccccgaccacatgaagcagcacgacttcttcaagtccgccatgcccgaaggctacgtccaggagcgcaccatcttcttcaaggacgacggcaactacaagacccgcgccgaggtgaagttcgagggcgacaccctggtgaaccgcatcgagctgaagggcatcgacttcaaggaggacggcaacatcctggggcacaagctggagtacaactacaacagccacaacgtctatatcatggccgacaagcagaagaacggcatcaaggtgaacttcaagatccgccacaacatcgaggacggcagcgtgcagctcgccgaccactaccagcagaacacccccatcggcgacggccccgtgctgctgcccgacaaccactacctgagcacccagtccgccctgagcaaagaccccaacgagaagcgcgatcacatggtcctgctggagttcgtgaccgccgccgggatcactctcggcatggacgagctgtacaagtaagtcgacaatcaacctctggattacaaaatttgtgaaagattgactggtattcttaactatgttgctccttttacgctatgtggatacgctgctttaatgcctttgtatcatgctattgcttcccgtatggctttcattttctcctccttgtataaatcctggttgctgtctctttatgaggagttgtggcccgttgtcaggcaacgtggcgtggtgtgcactgtgtttgctgacgcaacccccactggttggggcattgccaccacctgtcagctcctttccgggactttcgctttccccctccctattgccacggcggaactcatcgccgcctgccttgcccgctgctggacaggggctcggctgttgggcactgacaattccgtggtgttgtcggggaaatcatcgtcctttccttggctgctcgcctgtgttgccacctggattctgcgcgggacgtccttctgctacgtcccttcggccctcaatccagcggaccttccttcccgcggcctgctgccggctctgcggcctcttccgcgtcttcgccttcgccctcagacgagtcggatctccctttgggccgcctccccgcctggtacctttaagaccaatgacttacaaggcagctgtagatcttagccactttttaaaagaaaaggggggactggaagggctaattcactcccaacgaaaataagatctgctttttgcttgtactgggtctctctggttagaccagatctgagcctgggagctctctggctaactagggaacccactgcttaagcctcaataaagcttgccttgagtgcttcaagtagtgtgtgcccgtctgttgtgtgactctggtaactagagatccctcagacccttttagtcagtgtggaaaatctctagcagtagtagttcatgtcatcttattattcagtatttataacttgcaaagaaatgaatatcagagagtgagaggaacttgtttattgcagcttataatggttacaaataaagcaatagcatcacaaatttcacaaataaagcatttttttcactgcattctagttgtggtttgtccaaactcatcaatgtatcttatcatgtctggctctagctatcccgcccctaactccgcccagttccgcccattctccgccccatggctgactaattttttttatttatgcagaggccgaggccgcctcggcctctgagctattccagaagtagtgaggaggcttttttggaggcctagacttttgcagagacggcccaaattcgtaatcatggtcatagctgtttcctgtgtgaaattgttatccgctcacaattccacacaacatacgagccggaagcataaagtgtaaagcctggggtgcctaatgagtgagctaactcacattaattgcgttgcgctcactgcccgctttccagtcgggaaacctgtcgtgccagctgcattaatgaatcggccaacgcgcggggagaggcggtttgcgtattgggcgctcttccgcttcctcgctcactgactcgctgcgctcggtcgttcggctgcggcgagcggtatcagctcactcaaaggcggtaatacggttatccacagaatcaggggataacgcaggaaagaacatgtgagcaaaaggccagcaaaaggccaggaaccgtaaaaaggccgcgttgctggcgtttttccataggctccgcccccctgacgagcatcacaaaaatcgacgctcaagtcagaggtggcgaaacccgacaggactataaagataccaggcgtttccccctggaagctccctcgtgcgctctcctgttccgaccctgccgcttaccggatacctgtccgcctttctcccttcgggaagcgtggcgctttctcatagctcacgctgtaggtatctcagttcggtgtaggtcgttcgctccaagctgggctgtgtgcacgaaccccccgttcagcccgaccgctgcgccttatccggtaactatcgtcttgagtccaacccggtaagacacgacttatcgccactggcagcagccactggtaacaggattagcagagcgaggtatgtaggcggtgctacagagttcttgaagtggtggcctaactacggctacactagaaggacagtatttggtatctgcgctctgctgaagccagttaccttcggaaaaagagttggtagctcttgatccggcaaacaaaccaccgctggtagcggtggtttttttgtttgcaagcagcagattacgcgcagaaaaaaaggatctcaagaagatcctttgatcttttctacggggtctgacgctcagtggaacgaaaactcacgttaagggattttggtcatgagattatcaaaaaggatcttcacctagatccttttaaattaaaaatgaagttttaaatcaatctaaagtatatatgagtaaacttggtctgacagttaccaatgcttaatcagtgaggcacctatctcagcgatctgtctatttcgttcatccatagttgcctgactccccgtcgtgtagataactacgatacgggagggcttaccatctggccccagtgctgcaatgataccgcgagacccacgctcaccggctccagatttatcagcaataaaccagccagccggaagggccgagcgcagaagtggtcctgcaactttatccgcctccatccagtctattaattgttgccgggaagctagagtaagtagttcgccagttaatagtttgcgcaacgttgttgccattgctacaggcatcgtggtgtcacgctcgtcgtttggtatggcttcattcagctccggttcccaacgatcaaggcgagttacatgatcccccatgttgtgcaaaaaagcggttagctccttcggtcctccgatcgttgtcagaagtaagttggccgcagtgttatcactcatggttatggcagcactgcataattctcttactgtcatgccatccgtaagatgcttttctgtgactggtgagtactcaaccaagtcattctgagaatagtgtatgcggcgaccgagttgctcttgcccggcgtcaatacgggataataccgcgccacatagcagaactttaaaagtgctcatcattggaaaacgttcttcggggcgaaaactctcaaggatcttaccgctgttgagatccagttcgatgtaacccactcgtgcacccaactgatcttcagcatcttttactttcaccagcgtttctgggtgagcaaaaacaggaaggcaaaatgccgcaaaaaagggaataagggcgacacggaaatgttgaatactcatactcttcctttttcaatattattgaagcatttatcagggttattgtctcatgagcggatacatatttgaatgtatttagaaaaataaacaaataggggttccgcgcacatttccccgaaaagtgccacctgacgtctaagaaaccattattatcatgacattaacctataaaaataggcgtatcacgaggccctttcgtctcgcgcgtttcggtgatgacggtgaaaacctctgacacatgcagctcccggagacggtcacagcttgtctgtaagcggatgccgggagcagacaagcccgtcagggcgcgtcagcgggtgttggcgggtgtcggggctggcttaactatgcggcatcagagcagattgtactgagagtgcaccatatgcggtgtgaaataccgcacagatgcgtaaggagaaaataccgcatcaggcgccattcgccattcaggctgcgcaactgttgggaagggcgatcggtgcgggcctcttcgctattacgccagctggcgaaagggggatgtgctgcaaggcgattaagttgggtaacgccagggttttcccagtcacgacgttgtaaaacgacggccagtgccaagctg

**Lentivirus for miR-135a-5p knockdown：**

acgcgtgtagtcttatgcaatactcttgtagtcttgcaacatggtaacgatgagttagcaacatgccttacaaggagagaaaaagcaccgtgcatgccgattggtggaagtaaggtggtacgatcgtgccttattaggaaggcaacagacgggtctgacatggattggacgaaccactgaattgccgcattgcagagatattgtatttaagtgcctagctcgatacaataaacgggtctctctggttagaccagatctgagcctgggagctctctggctaactagggaacccactgcttaagcctcaataaagcttgccttgagtgcttcaagtagtgtgtgcccgtctgttgtgtgactctggtaactagagatccctcagacccttttagtcagtgtggaaaatctctagcagtggcgcccgaacagggacctgaaagcgaaagggaaaccagagctctctcgacgcaggactcggcttgctgaagcgcgcacggcaagaggcgaggggcggcgactggtgagtacgccaaaaattttgactagcggaggctagaaggagagagatgggtgcgagagcgtcagtattaagcgggggagaattagatcgcgatgggaaaaaattcggttaaggccagggggaaagaaaaaatataaattaaaacatatagtatgggcaagcagggagctagaacgattcgcagttaatcctggcctgttagaaacatcagaaggctgtagacaaatactgggacagctacaaccatcccttcagacaggatcagaagaacttagatcattatataatacagtagcaaccctctattgtgtgcatcaaaggatagagataaaagacaccaaggaagctttagacaagatagaggaagagcaaaacaaaagtaagaccaccgcacagcaagcggccactgatcttcagacctggaggaggagatatgagggacaattggagaagtgaattatataaatataaagtagtaaaaattgaaccattaggagtagcacccaccaaggcaaagagaagagtggtgcagagagaaaaaagagcagtgggaataggagctttgttccttgggttcttgggagcagcaggaagcactatgggcgcagcctcaatgacgctgacggtacaggccagacaattattgtctggtatagtgcagcagcagaacaatttgctgagggctattgaggcgcaacagcatctgttgcaactcacagtctggggcatcaagcagctccaggcaagaatcctggctgtggaaagatacctaaaggatcaacagctcctggggatttggggttgctctggaaaactcatttgcaccactgctgtgccttggaatgctagttggagtaataaatctctggaacagattggaatcacacgacctggatggagtgggacagagaaattaacaattacacaagcttaatacactccttaattgaagaatcgcaaaaccagcaagaaaagaatgaacaagaattattggaattagataaatgggcaagtttgtggaattggtttaacataacaaattggctgtggtatataaaattattcataatgatagtaggaggcttggtaggtttaagaatagtttttgctgtactttctatagtgaatagagttaggcagggatattcaccattatcgtttcagacccacctcccaaccccgaggggacccgacaggcccgaaggaatagaagaagaaggtggagagagagacagagacagatccattcgattagtgaacggatctcgacggtatcggttaacttttaaaagaaaaggggggattggggggtacagtgcaggggaaagaatagtagacataatagcaacagacatacaaactaaagaattacaaaaacaaattacaaaattcaaaattttatcgatgacattgattattgactagttattaatagtaatcaattacggggtcattagttcatagcccatatatggagttccgcgttacataacttacggtaaatggcccgcctggctgaccgcccaacgacccccgcccattgacgtcaataatgacgtatgttcccatagtaacgccaatagggactttccattgacgtcaatgggtggagtatttacggtaaactgcccacttggcagtacatcaagtgtatcatatgccaagtacgccccctattgacgtcaatgacggtaaatggcccgcctggcattatgcccagtacatgaccttatgggactttcctacttggcagtacatctacgtattagtcatcgctattaccatggtgatgcggttttggcagtacatcaatgggcgtggatagcggtttgactcacggggatttccaagtctccaccccattgacgtcaatgggagtttgttttggcaccaaaatcaacgggactttccaaaatgtcgtaacaactccgccccattgacgcaaatgggcggtaggcgtgtacggtgggaggtctatataagcagagctccgtttagtgaaccgtcagatcactagaagctttattgcggtagtttatcacagttaaattgctaacgcagtcagtgcttctgacacaacagtctcgaacttaagctgcagtgactctcttaaggtagccttgcagaagttggtcgtgaggcactgggcaggtaagtatcaaggttacaagacaggtttaaggagaccaatagaaactgggcttgtcgagacagagaagactcttgcgtttctgataggcacctattggtcttactgacatccactttgcctttctctccacaggaattcgccaccatggtgagcaagggcgaggagctgttcaccggggtggtgcccatcctggtcgagctggacggcgacgtaaacggccacaagttcagcgtgtccggcgagggcgagggcgatgccacctacggcaagctgaccctgaagttcatctgcaccaccggcaagctgcccgtgccctggcccaccctcgtgaccaccctgacctacggcgtgcagtgcttcagccgctaccccgaccacatgaagcagcacgacttcttcaagtccgccatgcccgaaggctacgtccaggagcgcaccatcttcttcaaggacgacggcaactacaagacccgcgccgaggtgaagttcgagggcgacaccctggtgaaccgcatcgagctgaagggcatcgacttcaaggaggacggcaacatcctggggcacaagctggagtacaactacaacagccacaacgtctatatcatggccgacaagcagaagaacggcatcaaggtgaacttcaagatccgccacaacatcgaggacggcagcgtgcagctcgccgaccactaccagcagaacacccccatcggcgacggccccgtgctgctgcccgacaaccactacctgagcacccagtccgccctgagcaaagaccccaacgagaagcgcgatcacatggtcctgctggagttcgtgaccgccgccgggatcactctcggcatggacgagctgtacaagcgtacgtgatagtaaggatcctcacataggaataaaaagccatatctggtggcggaggctcgggcggaggtgggtcgggtggcggcggatcatcacataggaataaaaagccatatctggtggcggaggctcgggcggaggtgggtcgggtggcggcggatcatcacataggaataaaaagccatatctggtggcggaggctcgggcggaggtgggtcgggtggcggcggatcatcacataggaataaaaagccatagtcgacaatcaacctctggattacaaaatttgtgaaagattgactggtattcttaactatgttgctccttttacgctatgtggatacgctgctttaatgcctttgtatcatgctattgcttcccgtatggctttcattttctcctccttgtataaatcctggttgctgtctctttatgaggagttgtggcccgttgtcaggcaacgtggcgtggtgtgcactgtgtttgctgacgcaacccccactggttggggcattgccaccacctgtcagctcctttccgggactttcgctttccccctccctattgccacggcggaactcatcgccgcctgccttgcccgctgctggacaggggctcggctgttgggcactgacaattccgtggtgttgtcggggaaatcatcgtcctttccttggctgctcgcctgtgttgccacctggattctgcgcgggacgtccttctgctacgtcccttcggccctcaatccagcggaccttccttcccgcggcctgctgccggctctgcggcctcttccgcgacttcgccttcgccctcagacgagtcggatctccctttgggccgcctccccgcctggtacctttaagaccaatgacttacaaggcagctgtagatcttagccactttttaaaagaaaaggggggactggaagggctaattcactcccaacgaaaataagatctgctttttgcttgtactgggtctctctggttagaccagatctgagcctgggagctctctggctaactagggaacccactgcttaagcctcaataaagcttgccttgagtgcttcaagtagtgtgtgcccgtctgttgtgtgactctggtaactagagatccctcagacccttttagtcagtgtggaaaatctctagcagtagtagttcatgtcatcttattattcagtatttataacttgcaaagaaatgaatatcagagagtgagaggaacttgtttattgcagcttataatggttacaaataaagcaatagcatcacaaatttcacaaataaagcatttttttcactgcattctagttgtggtttgtccaaactcatcaatgtatcttatcatgtctggctctagctatcccgcccctaactccgcccagttccgcccattctccgccccatggctgactaattttttttatttatgcagaggccgaggccgcctcggcctctgagctattccagaagtagtgaggaggcttttttggaggcctagacttttgcagagacggcccaaattcgtaatcatggtcatagctgtttcctgtgtgaaattgttatccgctcacaattccacacaacatacgagccggaagcataaagtgtaaagcctggggtgcctaatgagtgagctaactcacattaattgcgttgcgctcactgcccgctttccagtcgggaaacctgtcgtgccagctgcattaatgaatcggccaacgcgcggggagaggcggtttgcgtattgggcgctcttccgcttcctcgctcactgactcgctgcgctcggtcgttcggctgcggcgagcggtatcagctcactcaaaggcggtaatacggttatccacagaatcaggggataacgcaggaaagaacatgtgagcaaaaggccagcaaaaggccaggaaccgtaaaaaggccgcgttgctggcgtttttccataggctccgcccccctgacgagcatcacaaaaatcgacgctcaagtcagaggtggcgaaacccgacaggactataaagataccaggcgtttccccctggaagctccctcgtgcgctctcctgttccgaccctgccgcttaccggatacctgtccgcctttctcccttcgggaagcgtggcgctttctcatagctcacgctgtaggtatctcagttcggtgtaggtcgttcgctccaagctgggctgtgtgcacgaaccccccgttcagcccgaccgctgcgccttatccggtaactatcgtcttgagtccaacccggtaagacacgacttatcgccactggcagcagccactggtaacaggattagcagagcgaggtatgtaggcggtgctacagagttcttgaagtggtggcctaactacggctacactagaaggacagtatttggtatctgcgctctgctgaagccagttaccttcggaaaaagagttggtagctcttgatccggcaaacaaaccaccgctggtagcggtggtttttttgtttgcaagcagcagattacgcgcagaaaaaaaggatctcaagaagatcctttgatcttttctacggggtctgacgctcagtggaacgaaaactcacgttaagggattttggtcatgagattatcaaaaaggatcttcacctagatccttttaaattaaaaatgaagttttaaatcaatctaaagtatatatgagtaaacttggtctgacagttaccaatgcttaatcagtgaggcacctatctcagcgatctgtctatttcgttcatccatagttgcctgactccccgtcgtgtagataactacgatacgggagggcttaccatctggccccagtgctgcaatgataccgcgagacccacgctcaccggctccagatttatcagcaataaaccagccagccggaagggccgagcgcagaagtggtcctgcaactttatccgcctccatccagtctattaattgttgccgggaagctagagtaagtagttcgccagttaatagtttgcgcaacgttgttgccattgctacaggcatcgtggtgtcacgctcgtcgtttggtatggcttcattcagctccggttcccaacgatcaaggcgagttacatgatcccccatgttgtgcaaaaaagcggttagctccttcggtcctccgatcgttgtcagaagtaagttggccgcagtgttatcactcatggttatggcagcactgcataattctcttactgtcatgccatccgtaagatgcttttctgtgactggtgagtactcaaccaagtcattctgagaatagtgtatgcggcgaccgagttgctcttgcccggcgtcaatacgggataataccgcgccacatagcagaactttaaaagtgctcatcattggaaaacgttcttcggggcgaaaactctcaaggatcttaccgctgttgagatccagttcgatgtaacccactcgtgcacccaactgatcttcagcatcttttactttcaccagcgtttctgggtgagcaaaaacaggaaggcaaaatgccgcaaaaaagggaataagggcgacacggaaatgttgaatactcatactcttcctttttcaatattattgaagcatttatcagggttattgtctcatgagcggatacatatttgaatgtatttagaaaaataaacaaataggggttccgcgcacatttccccgaaaagtgccacctgacgtctaagaaaccattattatcatgacattaacctataaaaataggcgtatcacgaggccctttcgtctcgcgcgtttcggtgatgacggtgaaaacctctgacacatgcagctcccggagacggtcacagcttgtctgtaagcggatgccgggagcagacaagcccgtcagggcgcgtcagcgggtgttggcgggtgtcggggctggcttaactatgcggcatcagagcagattgtactgagagtgcaccatatgcggtgtgaaataccgcacagatgcgtaaggagaaaataccgcatcaggcgccattcgccattcaggctgcgcaactgttgggaagggcgatcggtgcgggcctcttcgctattacgccagctggcgaaagggggatgtgctgcaaggcgattaagttgggtaacgccagggttttcccagtcacgacgttgtaaaacgacggccagtgccaagctg
